# The Genomic Organisation of the TRA/TRD Locus Validates the Peculiar Characteristics of Dromedary δ-Chain Expression

**DOI:** 10.3390/genes12040544

**Published:** 2021-04-09

**Authors:** Serafina Massari, Giovanna Linguiti, Francesco Giannico, Pietro D’Addabbo, Salvatrice Ciccarese, Rachele Antonacci

**Affiliations:** 1 Department of Biological and Environmental Science and Technologies, University of Salento, 73100 Lecce, Italy; 2 Department of Biology, University of Bari “Aldo Moro”, 70125 Bari, Italy; giovanna.linguiti@uniba.it (G.L.); pietro.daddabbo@uniba.it (P.D.); salvatricemaria.ciccarese@uniba.it (S.C.); rachele.antonacci@uniba.it (R.A.); 3 Department of Veterinary Medicine, University of Bari “Aldo Moro”, 70010 Bari, Italy; francesco.giannico@uniba.it

**Keywords:** γδ T cell, T cell receptor, TRA/TRD locus, variable, diversity, joining and constant genes, complementarity determining region-3, δ chain, somatic hypermutation, Camelidae

## Abstract

The role of γδ T cells in vertebrate immunity is still an unsolved puzzle. Species such as humans and mice display a low percentage of these T lymphocytes (i.e., “γδ low species”) with a restricted diversity of γδ T cell receptors (TR). Conversely, artiodactyl species (i.e., “γδ high species”) account for a high proportion of γδ T cells with large γ and δ chain repertoires. The genomic organisation of the TR γ (TRG) and δ (TRD) loci has been determined in sheep and cattle, noting that a wide number of germline genes that encode for γ and δ chains characterise their genomes. Taking advantage of the current improved version of the genome assembly, we have investigated the genomic structure and gene content of the dromedary TRD locus, which, as in the other mammalian species, is nested within the TR α (TRA) genes. The most remarkable finding was the identification of a very limited number of variable germline genes (TRDV) compared to sheep and cattle, which supports our previous expression analyses for which the somatic hypermutation mechanism is able to enlarge and diversify the primary repertoire of dromedary δ chains. Furthermore, the comparison between genomic and expressed sequences reveals that *D* genes, up to four incorporated in a transcript, greatly contribute to the increased diversity of the dromedary δ chain antigen binding-site.

## 1. Introduction

The Camelidae family, belonging to the Artiodactyl order, comprises two groups of living camels. One, which resides in Northern Africa and Central Asia, consists of one-humped camels (*Camelus dromedarius*) and two-humped camels (*Camelus bactrianus* and *Camelus ferus*). The other group, the South American camelids, includes llama (*Lama glama*), guanaco (*Lama guanicoe*), alpaca (*Vicugna pacos*) and vicugna (*Vicugna vicugna*).

This small number of animal species has unique behavioural, anatomical and physiological characteristics compared to other species of artiodactyls, which have aroused great interest in the scientific community.

Camels are large herbivores and have a digestive system adapted to consume large amounts of different forages containing fibrous and thorny plants that are frequently found in the desert. This allows them to live under severe environmental conditions becoming essential for the economy and food security of local populations. Although they ruminate, distinct morphological features separate them from true ruminants such as the presence of a three-chambered foregut, different from the four-chambered foregut of sheep, cows and goats. Furthermore, camels have the ability to retain ingested material in the rumen longer than other ruminants, and the pH in camel rumen is closer to neutral, which supports lignocellulose degradation [1]. For this reason, camels are commonly called pseudo ruminants [2] and classified separately from true ruminants, in a family within Tylopoda, an Artiodactyla suborder.

Besides their adaptation to harsh environments, camels are multipurpose animals used for milk and meat production, racing, transportation and tourism.

Camels possess unusual features in the immune system [3,4]. In the context of the cellular-mediated response of the adaptive immunity, camels are similar to other artiodactyls, such as sheep, cows and pigs, with higher frequencies of blood γδ T cells (up to 35% of T cells) in younger animals. Therefore, camels belong to the “γδ high species”, in contrast to “γδ low species”, like humans and mice, where γδ T cells represent only a minor subpopulation (<5%) of circulating lymphocytes. Furthermore, it has been shown that in *C. dromedarius*, the rearranged variable domains of γ and δ chains of the T cell receptor (TR) display somatic mutations as a result of a mechanism that enhances the γδ T cell repertoire diversity [5,6,7].

The TR genes are multigene families organised into four TR loci distinct for each TR chain: TRA for α, TRB for β, TRG for γ and TRD locus for δ chain. The chains combine to form the heterodimeric receptor of the αβ or γδ T cells. Each TR locus consists of arrays of different gene types, including *variable* (*V*), *diversity* (*D*), *joining* (*J*) and *constant* (*C*) genes. Productive TRs are produced during the development of T lymphocytes in the thymus by somatic rearrangements of *V* and *J* genes in the TRA and TRG loci, and between *V*, *D*, and *J* genes in the TRB and TRD loci. After transcription, the resulting rearranged V–(D)–J region, encoding the variable domain of the TR chain, is spliced to the *C* gene, which encodes the constant domain of the receptor. The resulting TR chains are proteins with a variable domain at the N-terminal end, and a constant domain in the C-terminal end. Each variable domain comprises nine β sheets forming four framework regions or FR, which support three hypervariable loops (complementarity determining regions or CDR) [8,9]. Both CDR1 and CDR2 are encoded by the germline *V* gene; the third, CDR3, results from the V–(D)–J rearrangement.

The current development of improved versions of genome assemblies provides invaluable help for the study of complex genomic regions such as TR loci. In this context, the genomic organisation of the dromedary TRG locus has been recently completed by combining previous cloning data [6] with the analysis of genomic assemblies [10]. The locus is single with respect to ruminants’ TRG1 and TRG2 loci [11,12], and as in cows, sheep and goats it is organised in “cassettes”, each containing the basic V–J–J–C recombination unit. Only three cassettes with six functional TRGV germline genes compose the dromedary TRG locus. In contrast, the ruminant TRG loci, located in two distinct positions of the same chromosome, display a higher number of functional TRGV germline genes (eleven in sheep and goats, 16 in cattle) distributed, as in dromedary, in reiterated V–J–J–C cassettes (six in sheep and seven in cattle and goats). However, the reduced potential diversity of the dromedary TRG repertoire due to the lower number of germline genes is overcome thanks to the somatic hypermutation (SHM) mechanism that allows the expansion of the primary repertoire [6,7]. 

Similar to the γ chain, the ruminant as well as the pig T cell receptor δ chain repertoire is determined by a high number of germline genes, with a marked expansion and preferential usage of the TRDV1 multigene subgroup [13,14,15,16]. The recent annotation of the TRD genes [17], which are notoriously organised together with the TRA genes in a more complex TRA/TRD locus, revealed the existence up to 50 and 65 germline TRDV1 genes in cattle and in sheep respectively, compared with the single TRDV1 gene present in the human locus (IMGT^®^, http://www.imgt.org (accessed on 12 March 2021)). Four additional germline TRDV subgroups, nine TRDD and four TRDJ genes located in the TRD locus, contribute to the δ chain repertoire in these ruminant species.

In camel species, genomic data about the TRD locus are still poor and fragmented. It has been proposed that also the diversification of the δ chain repertoire in *C. dromedarius* is the result of SHM, particularly of the TRDV1 genes, which are poorly represented in the dromedary genome compared to the other artiodactyls [5]. The lack of solid information on the genomic organisation of the dromedary TRD locus has not allowed to substantiate this finding and, therefore some aspects of the δ repertoire could not be completely evaluated.

The latest release of a new high-quality version of dromedary reference genome assembly [18], which is chromosomally assigned and improved in terms of contiguity and increased size, prompted us to perform in-depth studies for understanding the genome architecture of the TRD locus that is critical for the discovery of the genetic basis of TR δ chain repertoire.

## 2. Materials and Methods

### 2.1. Genome Analysis

To determine the TRA/TRD locus location, the upgraded dromedary CamDro3 genome assembly [18] was searched using the BLAST algorithm. A sequence of 877,081 bp was retrieved directly from the reference sequence NC_044516.1 (*C. dromedarius* chromosome 6 genomic sequence) available at NCBI from 31,696,654 to 32,573,734 positions. Particularly, the analysed region extended from the TRAV1 to the TRAC, respectively, the first and the last gene of the TRA/TRD locus. The TRAV1 gene was located 6154 bp downstream of the *OR10G3* gene that borders different mammalian TRA/TRD loci studied so far, such as human, rhesus monkey, cat, rabbit and bovine loci. Likewise, the TRAC gene ends all the TRA/TRD loci at the 3′.

The available dromedary TRD germline gene sequences [5] as well as the human and sheep TRA/TRD genomic sequences (IMGT^®^, http://www.imgt.org (accessed on 12 March 2021)) [17] were used against the *C. dromedarius* genome sequence to identify, based on homology by the BLAST program, the corresponding genomic *V*, *D*, *J* and *C* genes. Moreover, the homology-based method was used, aligning the dromedary retrieved sequence against itself with the PipMaker program [19]. The beginning and end of each coding exon were identified with accuracy by the presence of splice sites or flanking recombination signal (RS) sequences of the *V*, *D* and *J* genes.

Locations of the TRA and TRD genes are provided in Supplementary Table S1. The locations of the *olfactory receptor* (*OR*) genes intermingled with the TRV genes at the 5′ of the locus are also provided (Supplementary Table S1).

Moreover, computational analysis of the dromedary TRA/TRD locus was conducted using the RepeatMasker for the identification of genome-wide repeats and low complexity regions (Smit, A.F.A., Hubley, R., Green, P. RepeatMasker open-4.0. at http://www.repeatmasker.org (accessed on 12 March 2021)) and Pipmaker [19] for the alignment of the determined dromedary sequence with itself. The inspection of the obtained dot-plot matrix allowed us to identify portions of the sequence that align with more regions within the sequence itself.

The CamBac2 and CamFer2 genome assemblies, which are the improved versions of the two Old World camelid genome sequences, and BCGSAC_Cfer_1.0 [18,20] were screened for the presence of the TRA/TRD region. A sequence from *TRAV1* to *TRAC* orthologous genes was retrieved from each assembly. Hence, the obtained sequences were compared with Mauve, a genome multiple alignment software available at http://darlinglab.org/mauve/mauve.html (accessed on 12 March 2021). For the genomic comparison among *C. dromedarius* (CamDro3), *C. bactrianus* (CamBac2) and *C. ferus* (CamFer2 and BCGSAC_Cfer_1.0) TRA/TRD regions, the dromedary sequences of the first and the last genes of the locus (*TRAV1* and *TRAC*, respectively pos. 31696654-31697280 and pos. 32569624-32573734 in NC_044516.1) were used to identify the corresponding sequences within CamBac2, CamFer2 and BCGSAC_Cfer_1.0 assemblies. CamBac2 and CamFer2 sequences are available in a fasta format from Dryad repository at https://doi.org/10.5061/dryad.qv9s4mwb3 (accessed on 12 March 2021). The wild Bactrian camel BCGSAC_Cfer_1.0 assembly is available from GenBank (GCA_009834535.1) and a sequence of approximately 605 kb was retrieved by similarity comparison in a chromosome 6 contig (NC_045701). CamBac2, CamFer2 and BCGSAC_Cfer_1.0 TRA/TRD sequences were multi-aligned with CamDro3 by MAUVE program (Multiple Genome Alignment, http://darlinglab.org/mauve/mauve.html (accessed on 12 March 2021)). The position of the annotated TRA/TRD genes was inserted in the dromedary sequence by using SnapGene utility (https://www.snapgene.com (accessed on 12 March 2021)).

### 2.2. Classification of the Dromedary TR Genes

The functional *V*, *D*, *J* and *C* genes were predicted through the manual alignment of sequences adopting the following parameters: (a) identification of the leader sequence at the 5′ of the *V* genes; (b) determination of proper RS sequences located at 3′ of the *V* (V–RS), 5′ and 3′ ends of the *D* (5′D-RS and 3′D-RS) and 5′ of the *J* (J-RS), respectively; (c) determination of conserved acceptor and donor splicing sites; (d) estimation of the expected length of the coding regions; (e) absence of frameshifts and stop codons in the coding regions of the genes. Conversely, a germline gene is qualified as ORF (open reading frame) if the coding region has an open reading frame, but alterations have described in the splicing sites and/or RS sequences, and/or in changes of conserved amino acids. Finally, a germline gene is qualified as pseudogene (P) if its coding region has stop codon(s) and/or frameshift mutation(s).

The TRAV and TRDV genes were grouped in different subgroups based on the percentage of nucleotide identity by using the Clustal Omega alignment tool, which is available at the EMBL-EBI website (http://www.ebi.ac.uk/ (accessed on 12 March 2021)), adopting the criterion that sequences with a nucleotide identity of more than 75% in the V-REGION (i.e., coding region of a TR *V* gene) belong to the same subgroup [21]. For some *V* pseudogenes exhibiting a nucleotide identity less than 75% in the V-REGION respect to some but not all member genes of its own subgroup, the entire nucleotide sequence, L-PART1 (exon encoding the first part of the leader peptide of a *V* gene), intron and V-EXON (germline sequence that comprises L-PART2 and V-REGION), was compared and the common structure with all subgroup genes, checked. The same structural analysis was applied to classify and/or assign to a subgroup, *V* pseudogenes with marked defects along the sequence.

Each subgroup was then classified by comparison with the sheep and/or human gene subgroups, taking into account the percentage of nucleotide identity, by means of BLAST program (https://blast.ncbi.nlm.nih.gov (accessed on 12 March 2021)) and the IMGT/V-QUEST tool [22]. Hence, based on the genomic position within the dromedary locus, each *TRAV* and *TRDV* gene was named following the IMGT nomenclature (http://www.imgt.org (accessed on 12 March 2021)).

The TRDD, TRDJ and TRAJ, and TRDC and TRAC genes were annotated and classified in accordance with the international nomenclature (IMGT^®^, http://www.imgt.org (accessed on 12 March 2021)) [23,24,25,26,27]. 

To confirm the presence of an additional TRDD gene, the TRDD region (from *TRDV2* to *TRDJ1*) was retrieved from *C. bactrianus* (CamBac2) and *C. ferus* (BCGSAC_Cfer_1.0) genomic assemblies and analysed compared with the corresponding region of the dromedary assembly (CamDro3). The comparison showed that the dromedary sequence was approximately 7.8 Kb shorter than the Bactrian one, with a gap between the TRDD4 gene and the TRDD5 gene where an additional TRDD gene in the Bactrian region is located. This gene had a sequence similar to the TRDD2 gene. The orthologous gene has been found on the chromosome 6 contig (NC_045701, pos. 31422287-31422298) of the wild Bactrian assembly BCGSAC_Cfer_1.0; whereas the same *TRDD* gene was not present within CamFer2 because of a gap.

### 2.3. Phylogenetic Analyses

The sheep and human TRAV, TRDV and TRDJ gene sequences used for the phylogenetic analyses were retrieved from the IMGT database (IMGT Repertoire, http://www.imgt.org (accessed on 12 March 2021)) [17,28]. 

The dromedary TRAV, TRDV and TRDJ genes were retrieved from the reference sequence NC_044516.1. 

Only one gene per each sheep and the human TRAV subgroup was included, first preferring potential functional genes when present. The human TRAV31 pseudogene, as a single member of its own subgroup, was excluded from the analysis because of several defects along the sequence. For the same reason, the dromedary TRAV8-3, TRAV16-1, TRAV16-2, TRAV19-4, TRAV22-5 and TRAV31 pseudogenes were not included in the tree.

Conversely, all dromedary and human genes were included in the TRDV tree, while only potential functional sheep TRDV genes and in-frame pseudogenes (except for the multimember TRDV1 subgroup genes indicated by “D”, duplicated genes, and “S”, provisional genes) were included.

We combined the nucleotide sequences of the V-REGION of the dromedary TRAV or TRDV genes with the corresponding gene sequences of sheep and human.

The phylogenetic relationship of the TRDJ genes were investigated by aligning the nucleotide sequences of all dromedary TRDJ genes (coding region plus RS) with those of sheep and humans.

Multiple alignments of the gene sequences under analysis were carried out with the MUSCLE program [29]. The evolutionary analyses were conducted in MEGAX [30,31]. We used the neighbour-joining (NJ) method to reconstruct the phylogenetic tree [32]. The evolutionary distances were computed using the p-distance method [33] and are in the units of the number of base differences per site.

## 3. Results

### 3.1. Genomic Organisation of the Dromedary TRA/TRD Locus Deduced from the Latest Version of the C. dromedarius Genome Assembly

**Figure 1:**
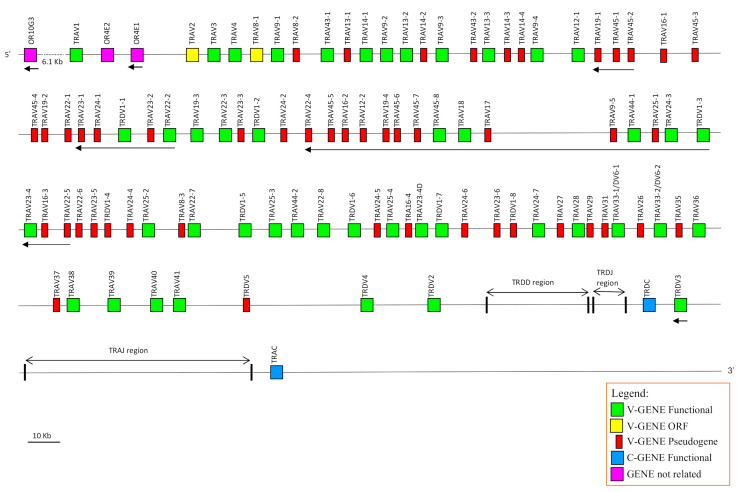
Schematic representation of the genomic organisation of the dromedary TRA/TRD locus deduced from the genome assembly CamDro3. The diagram shows the position of all related and unrelated TRA and TRD genes according to nomenclature. The boxes representing the genes are not to scale. The exons are not shown. The arrows indicate the transcriptional orientation of the genes.

The availability in the public database of a more accurate dromedary genome assembly (CamDro3) has prompted us to study in this camel species the genomic organisation of a complex locus such as the TRA/TRD. Thus, we retrieved a sequence of 877 kb in length from the *C. dromedarius* whole chromosome 6 genomic contig. The genomic sequence extended from the *TRAV1* gene, the first gene at the 5′ end of the locus embedded among three *OR* genes in conserved synteny with other mammalian species (IMGT^®^, http://www.imgt.org (accessed on 12 March)) [34], to the TRAC gene, which is located at the most 3′ end. The sheep and human corresponding genomic sequences (IMGT^®^, http://www.imgt.org (accessed on 12 March 2021)) [17] were used as a reference to identify and annotate all dromedary TRA and TRD genes. Basically, the deduced genomic structure of the dromedary TRA/TRD locus was found to be conserved with respect to the sheep and human as well as to the other species of mammals, with the TRD locus nested within the TRA locus. It includes, from 5′ to 3′, 81 TRAV and two TRAV/TRDV genes, intermingled with eleven TRDV genes, followed by six TRDD, four TRDJ, one TRDC and a single TRDV gene with an inverted transcriptional orientation (Figure 1). Besides the TRDV3 gene, 26 *V* genes lie in opposite orientation with respect to the transcriptional direction of the entire locus. At the 3′ end, the locus is completed with 60 TRAJ genes and one TRAC gene.

### 3.2. Classification, Structure and Phylogenetic Analysis of the TRAV Genes

The TRAV genes were assigned to 33 different subgroups by sequence analyses (see Section 2). Fifteen TRAV subgroups consist of more than one member genes with maximum expansion of the TRAV22 and TRAV45 (eight genes each), and the TRAV23 and TRAV24 (seven genes each). The TRAV9 subgroup consists of five genes, while the TRAV14, TRAV16, TRAV19 and TRAV25 subgroups contain four genes. Moreover, the TRAV8 and TRAV13 comprise three genes; and, finally, the TRAV12, TRAV33, TRAV43 and TRAV44 consist of two genes. It should be noted that the TRAV33 genes have been annotated as TRAV33/DV6 because they were found to rearrange with TRD(D)J genes within δ-chains productive cDNAs (see Section 3.6). Out of the 83 TRAV genes, 36 (approximately 45%) are predicted to be functional genes as defined by the IMGT rules (see Section 2; IMGT^®^, http://www.imgt.org (accessed on 12 March 2021)), and 47 (55%) are not functional (pseudogenes and ORF) (Table 1 and Supplementary Table S1). Most of the expanded TRAV subgroups consist predominantly of non-functional genes, with eight TRAV subgroups consisting of only pseudogenes. Therefore, the diversity of the functional germline repertoire is considerably reduced.

The deduced amino acid sequences of the potential functional germline TRAV genes, ORF and in-frame pseudogenes are shown in Supplementary Figure S1, where they are aligned according to IMGT unique numbering for the V-REGION [35] to maximise the percentage of identity. The TRAV gene subgroups showed a high structural diversity both in amino acid composition and in length. In particular, the CDR1-IMGT (definition according to the IMGT unique numbering for V-REGION) was five, six or seven amino acid lengths (AA) long. The CDR2-IMGT ranged in length from four to eight AA and the germline CDR3-IMGT was two to five AA long. Conversely, FR-IMGT varied above all in amino acid composition.

**Figure 2:**
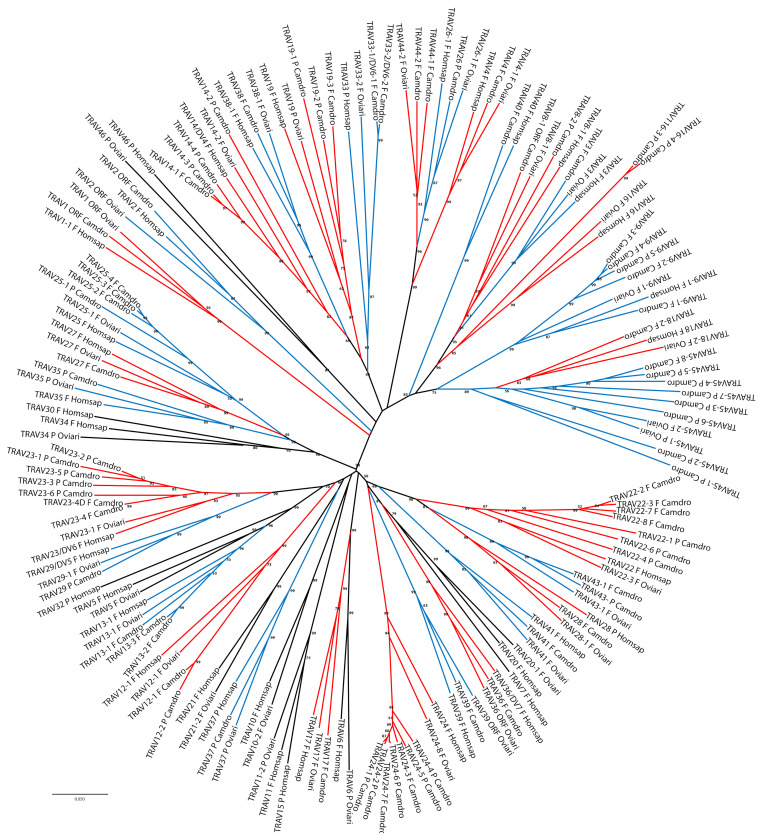
The neighbour-joining (NJ) tree inferred from the dromedary, sheep and human TRAV gene sequences. The evolutionary analyses were conducted in MEGA X [30,31]. The optimal tree with the sum of branch length = 19.33009914 is shown. The percentage of replicate trees in which the associated taxa clustered together in the bootstrap test (100 replicates) is shown next to the branches [36]. The tree is drawn to scale, with branch lengths in the same units as those of the evolutionary distances used to infer the phylogenetic tree. The evolutionary distances were computed using the p-distance method [33] and are in the units of the number of base differences per site. This analysis involved 159 nucleotide sequences. Codon positions included were 1st + 2nd + 3rd + Noncoding. All ambiguous positions were removed for each sequence pair (pairwise deletion option). There was a total of 405 positions in the final dataset. The orthologous TRAV genes formed monophyletic grouping that are alternatively highlighted in red and blue. The gene functionality according to IMGT rules (F: functional, ORF: open reading frame, P: pseudogene) is indicated. The IMGT 6-letter for species (Camdro, Oviari and Homsap) standardized abbreviation for taxon is used.

The classification and the membership of the dromedary TRAV genes to the subgroups have been validated by a phylogenetic analysis with the corresponding sheep and human genes. Sheep were chosen as representative of the order of artiodactyls in which the TRA/TRD locus has been well characterised and recently annotated [17], while the human germline genes represent the annotated reference repertoire. Adopting as the sole selection criterion the inclusion of only one sheep and human gene per subgroup, the V-REGION nucleotide sequences of all selected TRAV genes were combined in the same alignment and an unrooted phylogenetic tree was constructed using the NJ method [32] (Figure 2). 

The tree shows that each dromedary TRAV subgroup forms a monophyletic group with corresponding sheep and/or human genes, consistent with the birth of distinct subgroups prior to the divergence of the different species, with the exception of the TRAV45-1 and TRAV45-2 pseudogenes. Twelve TRAV subgroups are missing in the dromedary with respect to the human locus, and of these, four are missing also in the sheep locus (TRAV7, TRAV15, TRAV30 and TRAV32 subgroups). Moreover, three TRAV subgroups (TRAV43, TRAV44 and TRAV45) are shared between dromedary and sheep only; while the TRAV40 subgroup is missing in the sheep locus. Hence, the phylogenetic clustering confirmed the previous classification by sequence analysis of each dromedary TRAV genes as orthologous to the corresponding sheep and human genes.

### 3.3. Classification, Structure and Phylogenetic Analysis of the TRDV Genes

Twelve TRDV genes were assigned to five different subgroups by sequence analyses (see Section 2). Only the TRDV1 is a multimember gene subgroup with eight genes that are intermingled with the TRAV genes. The other subgroups (TRDV2, TRDV3, TRDV4 and TRDV5) consist of one member gene each, which are located after the TRAV gene pool, within the TRD locus (Figure 1). Three out of twelve TRDV genes (25%) have been predicted to be pseudogenes (Supplementary Table S1).

**Figure 3:**
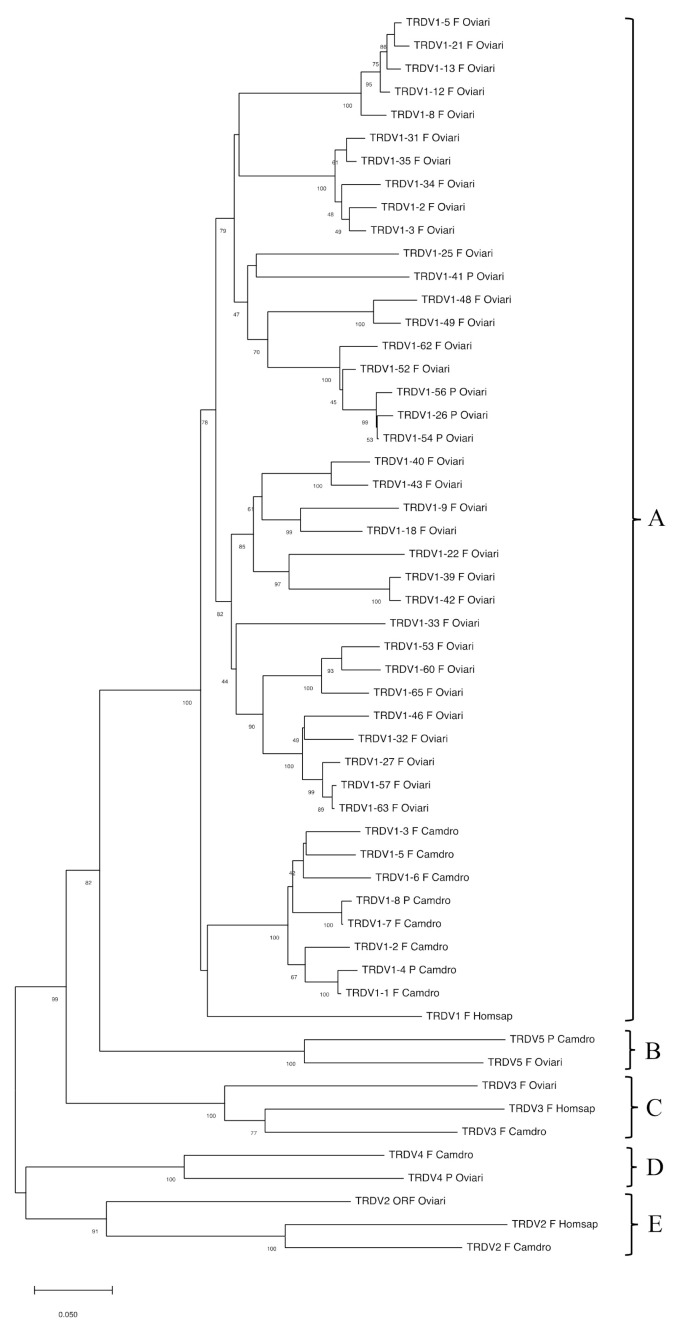
The NJ tree inferred from the dromedary, sheep and human TRDV gene sequences. The five groups (A–E) of the TRDV genes drawn by the tree are indicated by brackets. The evolutionary analyses were conducted in MEGA X [30,31]. The optimal tree with the sum of branch length = 4.03998833 is shown. The percentage of replicate trees in which the associated taxa clustered together in the bootstrap test (100 replicates) are shown below the branches [36]. The tree is drawn to scale, with branch lengths in the same units as those of the evolutionary distances used to infer the phylogenetic tree. The evolutionary distances were computed using the p-distance method [33] and are in the units of the number of base differences per site. This analysis involved 54 nucleotide sequences. Codon positions included were 1st + 2nd + 3rd + Noncoding. All ambiguous positions were removed for each sequence pair (pairwise deletion option). There was a total of 411 positions in the final dataset. The gene functionality according to IMGT rules (F: functional, ORF: open reading frame, P: pseudogene) is indicated. The IMGT 6-letter for species (Camdro, Oviari and Homsap) standardized abbreviation for taxon is used.

The classification of the dromedary TRDV subgroups was established by comparing all dromedary genes with available corresponding genes in sheep and humans (Figure 3).

Group B and D are representative of genes belonging to artiodactyls as they include dromedary and sheep TRDV5 and TRDV4 genes, respectively.

In C, the dromedary, sheep and human TRDV3 genes are grouped, that stand in an inverted transcriptional orientation downstream the TRDC gene (Figure 1) (IMGT^®^, http://www.imgt.org (accessed on 12 March 2021)) [17]. Finally, group E contains the TRDV2 corresponding genes of all the three species.

Supplementary Figure S2 shows the protein display of the dromedary TRDV genes. The deduced amino acid sequences of the functional genes were manually aligned according to IMGT unique numbering for the V-REGION [35]. The TRDV gene subgroups show a structural diversity essentially in amino acid composition. The amino acid lengths of the CDR-IMGT are identical for all genes (7.3.4) except for the CDR2-IMGT of the TRDV3 gene (three AA longer) and for the germline CDR3-IMGT of the TRDV2 (one AA shorter).

### 3.4. Architecture of the Dromedary V-CLUSTER Containing Region

**Figure 4:**
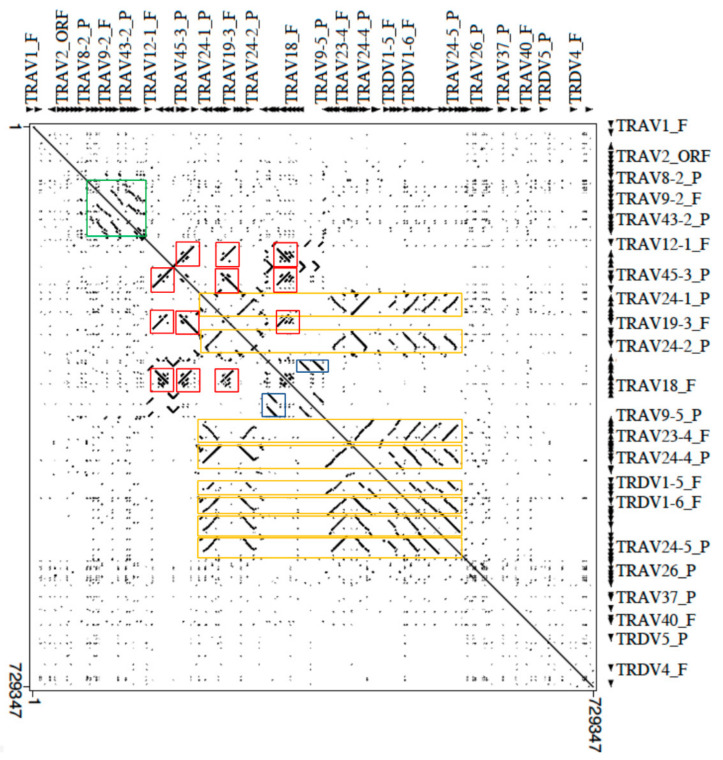
Dot-plot matrix of the dromedary V-cluster sequence against itself. With the exception of the main diagonal line for the match of each base with itself, dots and lines indicate internal homology units in the sequence. The colored boxes show that the V region underwent to duplication events (see text). Orthogonal lines indicate units of homology between regions with an opposite orientation.

The genomic structure of the V-CLUSTER of the dromedary TRA/TRD locus was investigated aligning the masked corresponding sequence (from TRAV1 to TRDV2 gene) against itself with the Pipmaker program (Figure 4).

Legend of the colored boxes: orange for TRDV1 genes; red for TRAV19 and TRAV45 genes; green for TRAV9, TRAV13, TRAV14 and TRAV43 genes; blue for TRAV45 genes.

The dot-plot matrix highlights the high level of nucleotide identity between V genes as indicated by dots and lines. Furthermore, lines parallel and orthogonal to the perfect main diagonal line, which indicates the match of each base of the sequence with itself, detect units of internal homology due to the duplicative events that have arisen within the multimember TRV genes subgroups. Notable features are the multiple homology regions (parallel or perpendicular lines depending on the orientation of regions within the genomic sequence) due to the expansion of the genes (eight members) of the TRDV1 subgroup (eight orange rectangles in Figure 4). Each homology lines also includes members of expanded TRAV subgroups intercalated with the TRDV1 genes: TRAV22 (eight copies), TRAV23 and TRAV24 (seven copies) and TRAV25 (four copies) subgroups. In fact, the longest homology unit, of about 24 kb (TRAV22-6/TRAV23-5/TRDV1-4/TRAV24-4/TRAV25-2) contains member genes of all these subgroups. Therefore, the TRDV1 amplification mechanism may have facilitated the generation of multigene subgroups even between TRAV genes or vice versa. Such concomitant expansion of the TRDV1 and TRAV genes has been also shown in the sheep locus where the TRDV1 genes are always co-localised with genes of expanded TRAV subgroups, mostly belonging to corresponding dromedary TRAV subgroups [17,34].

Similarly, parallel or perpendicular lines, in the dot, identify duplication areas in which the TRAV19 subgroup genes have arisen through duplication events which have involved also the TRAV45 subgroup (red boxes in Figure 4), generating TRAV19–TRAV45–TRAV45 blocks.

Finally, parallel lines that identify multiple tandem duplications of an internal homology unit consisting of the TRAV9, TRAV13, TRAV14 and TRAV43 (green box in Figure 4) subgroup genes and parallel lines due to the tandem duplication of the TRAV45 genes (blue boxes in Figure 4) were observed.

### 3.5. Description of the D, J and C Genes at the 3′ Region of the TRA/TRD Locus

Following the V-CLUSTER, the TRA/TRD locus continues with the TRD genes (Figure 1 and Supplementary Figure S3). Six TRDD genes were localised in a region spanning about 49 kb between the TRDV2 and the first TRDJ gene. To date, nine TRDD genes in a region of about 112 kb have been demonstrated in sheep TRD locus [17], whereas only three TRDD genes lie in the human region of about 27 kb (IMGT^®^, http://www.imgt.org (accessed on 12 March 2021)). The nucleotide and deduced amino acid sequences of the dromedary TRDD genes identified in the region are shown in Supplementary Figure S4A. They were annotated and classified according to the international nomenclature (IMGT^®^, http://www.imgt.org (accessed on 12 March 2021)), [23,24] with a number corresponding to their position from 5′ to 3′ within the locus. The six TRDD genes consist of a 9 (TRDD3), 11 (TRDD2 and TRDD6) and 13 bp (TRDD1, TRDD4 and TRDD5) sequence that can be productively read through their three coding phases. The 5′D–RS and 3′D–RS sequences that flank the D-REGION (i.e., coding region of a TR D gene) are well conserved with respect to the consensus sequence except for the non-canonical RS of TRDD3 3′ heptamer, where the C nucleotide located in the third nucleotide position is mutated to T.

Consistent with sheep and humans (IMGT^®^, http://www.imgt.org (accessed on 12 March 2021)) [17], four TRDJ genes are located upstream of the TRDC gene. The nucleotide and deduced amino acid sequences of the TRDJ genes are shown in Supplementary Figure S4B. They are typically 49–60 bp long. Each TRDJ gene is flanked by the J–RS at the 5′ end, and a donor splice site at the 3′ end. The coding regions were all predicted to be functional and conserve the canonical F–G–X–G amino acid motif, whose presence characterises the functional J genes. The dromedary TRDJ genes were classified in accordance with their high nucleotide identity with the sheep and human orthologues. A phylogenetic tree confirms the evolutionary relationship between the clustering distribution of the corresponding TRDJ genes in the three species and their order within each TRD locus (Supplementary Figure S5).

The exon–intron organisation of the TRDC gene was also determined (Supplementary Figure S4C). The dromedary TRDC gene encodes a protein of 152 AA. The human TRDC gene encodes a protein of 155 AA, while the sheep protein is 156 AA long (IMGT^®^, http://www.imgt.org (accessed on 12 March 2021)) [17]. As in the other mammalian species, it is composed of three translated exons plus a fourth untranslated one. The C-DOMAIN is encoded by EX1 and is 93 AA long as in sheep and humans. The C region also comprises the connecting region (CO) of 34 AA (encoded for 22 AA by EX2 as in human, while in sheep EX2 encodes for 25 AA, and for 12 AA by EX3) with a cysteine involved in the interchain disulphide bridge, the transmembrane (TM) of 20 AA (encoded by the 3′ part of EX3) and the cytoplasmic region (CY) of five AA (encoded by the last part of EX3).

The transcriptionally inverted TRDV3 gene ends the TRD locus. Sixty TRAJ genes lie in the genomic region between TRDV3 and TRAC genes (Supplementary Figures S3 and S6A). Fifty-five TRAJ genes were assessed as functional genes and they conserve the canonical F/W–G–X–G amino acid motif (where F is phenylalanine, W tryptophan, G glycine and X any AA) (IMGT^®^, http://www.imgt.org (accessed on 12 March 2021)) [37]. Each TRAJ gene is flanked by the J–RS at the 5′ end, and a donor splice at the 3′ end. The dromedary TRAJ genes were named on the basis of their homology with sheep and bovine genes [17].

The TRAC gene surrounds the TRA/TRD locus (Supplementary Figures S3 and S6B). The dromedary TRAC gene consists of three translated exons and a fourth untranslated exon. The first, second and third exons are 261, 45 and 108 bps in length, respectively. The fourth exon is 573 bp. These four exons are separated by introns that are 1456, 1008 and 660 bps in length, respectively. The exons encode a protein of 121 AA. The C-DOMAIN is encoded by EX1 and is 87 AA long. The C region also comprises the connecting region (CO) of 27 AA (encoded for 15 AA by EX2 and for 12 AA by EX3) with a cysteine involved in the interchain disulphide binding, the transmembrane (TM) of 20 AA (encoded by the 3′ part of EX3) and the cytoplasmic region (CY) of three AA (encoded by the last part of EX3).

### 3.6. Relationship of the Dromedary TRDV Germline Genes with Public Available Gene Sequences and Analysis of the δ-Chain Repertoire

A suggestive implication of the dromedary TRA/TRD gene annotation is represented by the possibility of a detailed assessment of the functional δ chain repertoire earlier determined in *C. dromedarius* [5]. By a combination of rapid amplification of cDNA ends (RACE) and reverse transcription-polymerase chain reaction (RT-PCR), Antonacci and colleagues [5] evaluated the expressed δ chain repertoire and identified TRDV genes classified into three distinct subgroups: TRDV1, TRDV2 and TRDV4. Hence, δ chain transcripts were used to identify germline TRDV genes in the dromedary genome by genomic PCRs. Six distinct functional TRDV1, two TRDV2 genes and one TRDV4 gene were isolated. The amount of TRDV germline genes, especially the low number of TRDV1, in the dromedary genome was then demonstrated by real-time PCR (qPCR) data, too. In the present work, the characterisation of the TRD genes in the dromedary genome assembly has showed the presence of eight TRDV1 genes (six functional genes and two pseudogenes), which is perfectly in line with the data already described [5]. We have aligned the nucleotide sequence of the two TRDV1 gene groups: six functional germline genes retrieved from the genome assembly with six distinct TRDV1 genes isolated before. The comparison between the two groups of TRDV1 genes allowed us to establish that five genes perfectly match each other (Table 2), based on an operational criterion according to which sequences sharing >97% of nucleotide identity represent the same genes.

Otherwise, two genes, the TRDV1-1 gene annotated within the locus and the TRDV1-5 gene [5], showed in the comparison a range of percentage of nucleotide identity <97%, indicating that the TRDV1-1 could represent a distinct gene not found in the previous analysis, while the absence of the corresponding TRDV1-5 gene in the TRA/TRD locus would suggest the presence of a gap in the current assembly. However, polymorphisms or sequencing errors affecting for instance the nucleotide sequence of the TRDV1 pseudogenes, cannot be ruled out.

Similarly, we aligned the nucleotide sequences of the two TRDV2 genes and the single TRDV4 gene previously isolated [5] with the gene sequences annotated in this study in order to find any correspondences between them. The comparison showed that the two genes, previously named TRDV2 (TRDV2-1 and TRDV2-2) (Table 2) because of their homology with the corresponding sheep sequence [13], actually correspond to the TRAV33 genes (TRAV33-1 and TRAV33-2). As these genes, structurally belonging to the group of TRAV genes, have been found rearranged with TRDD and/or TRDJ genes and therefore used in the synthesis of δ chains in sheep [13] as well as in dromedary [5], they have been classified as TRAV33/DV6 (Table 2 and Figure 1).

Finally, the TRDV4 gene, classified according to the initial sheep nomenclature [16], perfectly matches the TRDV3 gene that stands in an inverted transcriptional orientation downstream of the TRDC gene within the TRA/TRD locus (Table 2 and Figure 1).

Definitively, our genomic analysis confirms the substantial difference in regard to the number of the TRDV1 germline genes observed when dromedary is compared with the other artiodactyl species [16,17,38,39].

Despite the somatic mutation, we tentatively evaluated the contribution of each annotated germline genes in the formation of the δ chain repertoire with particular reference to the CDR3-IMGT region. A total of 61 cDNA clones isolated from peripheral lymphoid tissues of an adult healthy dromedary (22 from spleen, 15 from tonsils and 24 from blood) and containing unique rearranged productive V–(D)–J–C transcripts, were selected from the cDNA library [5] and analysed.

Although nucleotide changes with respect to the germline sequences, 39 cDNA clones (nine in spleen, eight in tonsils and 22 in blood) consist of a member of the TRDV1 subgroup, 11 clones (seven in spleen and four in tonsils) contain the TRAV33/DV6 genes and 11 clones (six in spleen, three in tonsils and two in blood) retain the TRDV3 gene.

The comparison between germline and cDNA sequences has also allowed us to establish that all annotated TRDJ genes are represented, with the germline TRDJ1 gene corresponding to the TRDJ5 classified within the cDNAs [5].

For a close inspection of the enlarged CDR3-IMGT region, the nucleotide sequences from codon 105 (codon following the 2nd-CYS codon 104 of the V-REGION) to codon 117 (codon preceding the J–PHE 118 or J–TRP 118) that belongs to the F/W–G–X–G motif characteristic of the J–REGION (i.e., coding region of a J gene) (IMGT^®^, http://www.imgt.org (accessed on 12 March 2021)), was excised from each clone and reported in Supplementary Figure S7. By comparison with the TRDD genomic sequences, the nucleotides located in each sequence were considered to belong to a TRDD gene if they constituted a stretch of at least five consecutive nucleotides corresponding to one of the six TRDD germline sequences. All TRDD but one (TRDD3) gene, with no preference in the use, were recovered within the cDNA clones. Moreover, in 12 clones the presence of an additional TRDD gene (TRDD* in Supplementary Figure S7) with a nucleotide sequence similar to the TRDD2 and missing in the current genome assembly has been hypothesized. We proved the presence of this additional TRDD gene with an analysis aimed at the TRDD region in *C. Bactrianus* (CamBac2) and *C. ferus* (BCGSAC_Cfer_1.0) genome sequences, which revealed the presence of seven TRDD genes, indicating a probably gap between the dromedary TRDD4 and TRDD5 where one more TRDD similar to the TRDD2 gene would lie (see Section 2).

Hence, we observed four clones with no recognizable TRDD gene that could be interpreted as a direct V–J junction. However, it is also possible that nucleotide trimming as well as somatic mutation masked the participation of the TRDD gene during the rearrangement. Nevertheless, the presence of an unidentified TRDD gene in the genome assembly cannot be excluded. In 14 clones, there is the presence of a single TRDD gene, while 20 and 17 clones have two or three TRDD genes, respectively. Six clones even contain four TRDD genes.

In some cases, two or three or four TRDD genes are continuous, in others they are separated by P/N nucleotides. As expected, the order of the TRDD, within the CDR3 junction with more than one gene, corresponds to that found in the genome.

The mean length of CDR3-IMGT is 20.4 AA (range 9–37 AA) without a significant difference in the three tissues but with an appreciable increase in the length in clones of the TRDV1 subgroup compared to the TRAV33/DV6 and especially the TRDV3 (Table 3).

The CDR3-IMGT length seems to be correlated to the number of the TRDD genes incorporated in the loop (Table 4). 

In fact, when we analysed the CDR3-IMGT region of all except four clones in which it was not possible to recognize any TRDD gene, we found a progression in the CDR3-IMGT length from clones with one TRDD (mean 18.6 AA, range 9–27 AA) and two (mean 19.2 AA, range 12–28 AA) and three TRDD genes (mean 21.1 AA, range 15–28 AA), reaching a maximum of the CDR3-IMGT length (mean 28.3 AA, range 18–37) in the clones with four TRDD genes. It should be noted that the CDR3-IMGT, consisting of four TRDD genes, was found only in clones of the TRDV1 subgroup.

However, in the progression of the CDR3-IMGT length, the increase of the number of TRDD genes is accompanied by the decrease of P/N additions (Supplementary Figure S8). Therefore, the insertion of P/N nucleotides does not seem to adhere to any specific rules, but rather it appears to act to ensure appropriate length of the CDR3 for δ chain.

### 3.7. Comparative Analysis of the TRA/TRD Locus in the Camelus Genus

The dromedary TRA/TRD sequence was used as a reference for an overview, by computational method, of the orthologous regions in three genome assemblies (CamBac2, CamFer2 and BCGSAC_Cfer_1.0) of the most closely related species available in databases, i.e., *C. bactrianus* and *C. ferus*.

The CamDro3 chr6:31696654-32573734 region (877081 bp) matched with CamBac2 chr6:29891560-30751197 region (859638 bp), CamFer2 chr6:30383898-31277736 region (893839 bp), and a very short region in a contig of BCGSAC_Cfer_1.0 (NC_045701:30928671-31534193).

**Figure 5:**
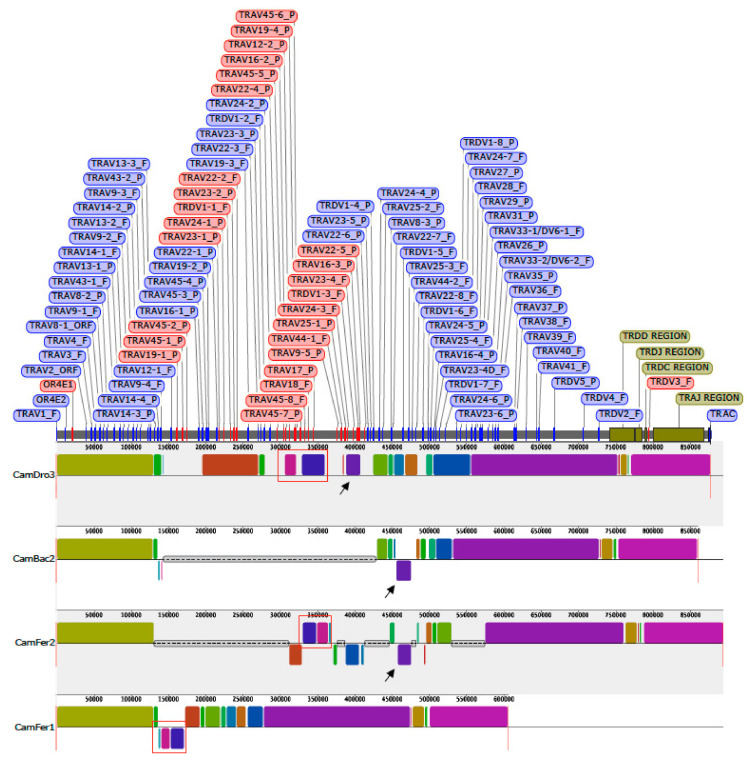
Schematic representation of the comparison of the dromedary (CamDro3) TRA/TRD genomic region with Bactrian (CamBac2) and wild Bactrian (CamFer2 and CamFer1) sequences. Colored blocks highlight syntenic regions between the different assemblies. Blocks below the line indicate sequences of inverted orientation with respect to the reference CamDro3. The grey thin boxes represent sequencing gaps. The position of all dromedary TRA and TRD genes is reported. The genes with an opposite orientation within the dromedary TRA/TRD locus are marked in red. The black arrows and the red boxes are described in the text.

A multiple-alignment analysis identified conserved blocks of synteny within each assembly, as presented in Figure 5. 

The distribution of the corresponding blocks, indicated by identical colors, reveals the wide co-linearity between the different TRA/TRD sequences. This is in accordance with previous comparative genomic analysis that reveals the existence of a co-linearity between the three Old World camel TRB genomic sequences [40]. Indeed, large blocks of synteny are interrupted by shortest blocks, due to the presence of sequencing gaps (grey lines in Figure 5) or micro rearrangements (e.g., local inversion, blocks below the central line of each graphic in Figure 5) in some of the three genomic sequences. Taking into account that the present gaps are rightly estimated in size, the length of the entire TRA/TRD locus appears to be comparable in the recent draft of the genome sequences belonging to the three camel species [18]. Instead, sequence gap annotation is lacking in the assembly BCGSAC_Cfer_1.0 of the wild Bactrian camel [20].

Mostly, the sequencing gaps affect the regions where the TR V genes have been found to be in opposite orientation with respect to the 5′–3′ direction of the locus. It is possible that the presence of regions with duplicated genes organised in an inverted transcriptional orientation makes it difficult to assemble them in a continuous genomic sequence and, therefore, inconsistencies and sequencing gaps are more frequent. For instance, black arrows in Figure 5 indicate that the corresponding purple blocks, consisting of dromedary TRAV24-3, TRDV1-3, TRAV23-4 and TRAV16-1 genes, are not located in a syntenic region in CamBac2 as well as in CamFer2 sequences. Moreover, both in CamBac2 and CamFer2, the purple block has been assembled in an opposite orientation with respect to the CamDro3 one. Finally, the corresponding purple block is completely missing within the other *C. ferus* sequence (BCGSAC_Cfer_1.0), showing that the CamFer2 assembly represents an improved version also for the TRA/TRD region.

Similarly, the blocks containing the *C. dromedarius* inverted genes, from TRAV22-4 to TRAV17 (red boxes in Figure 5), are lacking in CamBac2 but are present in CamFer2 as well as in BCGSAC_Cfer_1.0, even if the orientation in the last assembly is different.

## 4. Discussion

In this work, the latest improved version of the genomic assembly [18] allowed us to fill the gap in our knowledge [5] regarding the genomic organisation of the dromedary TRA/TRD locus, which represents the most complex among the TR loci. 

As in all mammalian species studies so far, the dromedary TRA/TRD locus has the common feature of the presence of TRD genes intermingled with the TRA genes. The dromedary TRA/TRD locus spans about 870 Kb long, making it smaller in size with respect to the homologous region of human (1000 Kb) and sheep (2882 Kb) (IMGT^®^ Repertoire, http://www.imgt.org (accessed on 12 March 2021)) [17]. These two species were considered for the comparison, taken first as a reference sequence, and second as being representative of the artiodactyl species.

Although the general structure is shared between the three species, interesting differences in the gene content can be observed. In dromedary, the total number of TRAV genes (83 TRAV genes) is higher than in humans (56 TRAV genes) (IMGT^®^ Repertoire, http://www.imgt.org (accessed on 12 March 2021)), but is much lower than in the sheep (277 TRAV genes) [17]; whereas the number of the dromedary TRAV subgroups (33 subgroups) is lower with respect to both humans (42 subgroups) and sheep (39 subgroups). Therefore, the dromedary germline TRAV repertoire is mainly due to the complexity of the duplication events that have caused the expansion of genes within some subgroups rather than the birth and/or the maintenance of other subgroups. As a matter of a fact, the clustering of the genes in the phylogenetic tree shows that gene duplication, involving ancestral TRAV gene subgroups followed by diversification, is the major mode of evolution of the dromedary TRAV genes. However, the dromedary TRAV42, TRAV43 and TRAV45 subgroups, shared with sheep but not with humans, are distinctive of the artiodactyl lineage, only.

It is possible that the expansion in the dromedary genome of some multigene TRAV subgroups could be in part the direct consequence of the gene expansion of the TRDV1 subgroup. How the mechanism of TRDV1 amplification could have also facilitated the generation of multigene TRAV subgroups is evident in the dot-plot matrix. A noticeable feature is the presence in the central part of the matrix of multiple homology units, due to the expansion of the eight dromedary TRDV1 subgroup members together with genes of the TRAV22, TRAV23, TRAV24 and TRAV25 subgroups. The corresponding TRAV subgroups, which consist of only one member gene, is flanked by the single TRDV1 gene in the human TRA/TRD locus (IMGT^®^ Repertoire, http://www.imgt.org (accessed on 12 March 2021)), indicating that the TRAV22-TRAV23/DV-TRDV1-TRAV24-TRAV25 region represents an ancestral block. The concomitant expansion of TRDV and TRAV gene repertoires appears to be occurred also in the sheep locus [17,34] where the expansion of 65 members of the TRDV1 subgroup involved more TRAV subgroups including the corresponding dromedary, too. As a direct consequence of the wide TRAV expansion, it is the presence, in both species, of a high level (55%) of not-functional TRAV genes, mostly belonging to the expanded TRAV subgroups. Differently, in humans, only 32% of TRAV genes are not functional.

The dromedary matrix also highlights the presence within the V cluster of extensive inversion regions also exhibited in the sheep as well as in other ruminant TRA/TRD locus [17].

The dromedary TRA locus is completed by 60 TRAJ genes and one TRAC gene, similar to humans but different to sheep, where 61 and 79 TRAJ genes were found, respectively.

In the dromedary TRD locus, twelve germline TRDV genes, distributed in five subgroups, were identified. Only eight members with respect to the high number in sheep as well as in other artiodactyls [16,17,38,39], belong to the TRDV1 subgroups. Six out of eight TRDV1 are functional genes and present a high level of nucleotide identity (from 93% to 97%), with a low level of structural diversity, similar length and amino acid composition in the CDR. Differently, in sheep, 65 TRDV1 subgroup genes are largely diversified by a structural variability that includes increased area of CDR and a high degree of amino acid variations, with a range of nucleotide identity from 78 to 97% [16]. The diversification at the CDR has probably guaranteed the maintenance of functional multiple copies of the genes of the TRDV1 subgroup in this species.

Hence, our data confirm that, given the low number of TRDV1 germline genes in the dromedary locus, the SHM process plays a key role in the generation of the δ chain repertoire in this species, allowing it to achieve diversity comparable to that of sheep [5].

The remaining dromedary TRDV subgroups (TRDV2, TRDV3, TRDV4 and TRDV5) consist of a single gene each, with the TRDV5 gene classified as a pseudogene. The phylogenetic approach demonstrated that TRDV4 and TRDV5 genes are typical of the artiodactyl species, while TRDV2 is shared with humans. In this regard, we did not find the presence of spleen transcripts encoding TRDV2 chains as demonstrated in cDNAs from the blood of the New World camelid alpaca (Vicugna pacos) [41]. The TRDV2 gene is involved in the formation of Vγ9Vδ2 T cells, the major γδ T cell subset in human blood. These cells were demonstrated to be important mediators of immunosurveillance and targets for cell-based immunotherapy and the alpaca represents the prime candidate for the first non-primate species in which Vγ9Vδ2 T cell subset was demonstrated.

Moreover, the phylogenetic tree also attests that the gene duplications that affected the birth of the expanded TRDV1 subgroup occurred independently in sheep and dromedary genome, although the model of duplication can be the same as indicated by the dot-plot matrix information.

The TRDD genes greatly contribute to the dromedary δ chain repertoire since six or more probably seven TRDD genes are capable of generating many potential V–(D)–J recombinations similar to sheep (nine TRDD genes) rather than to humans (three TRDD genes). Differently, dromedary, sheep and humans share the same number of TRDJ genes. Therefore, TRDD genes strongly participate in the diversity of dromedary δ CDR3. Evidence [42,43] suggests that γδ T cell antigen specificity lies predominantly in the CDR3 loop of the TR δ chain. Our cDNA analysis revealed that the length of dromedary δ CDR3 (means length 20.4 AA; range 9–37 AA) mainly depends on the incorporation of more sequential TRDD genes during the recombination process rather than the reduced level of trimming at the ends of the TRDV and TRDJ genes. The maximum number of incorporated TRDD genes is 4 in 6 out of 61 clones, with CDR3 mean length of 28.3AA (range 18–37).

By comparison, in sheep, the mean length of δ CDR3 is 17.15 AA (range 8–26 AA) with only one clone out of 56 containing four TRDD genes [34]. Similarly, pig δ chains can involve up to four TRDD genes [39], and the cattle δ CDR3 shows combinations from one up to five TRDD genes [38].

Differently, looking at the β CDR3 also formed by V–D–J junctions, the mean length is 12.8 AA (range 9–19 AA) in dromedary spleen, with one clone out of 35 containing two TRBD genes, although there was a different genomic organisation of the TRB locus [44]. A similar length was reported in sheep spleen β CDR3 that is 13.18 AA long (range 9–20 AA) [45]. Rarely, two TRBD genes are also present in the pig β CDR3 region [46], where the mean length of β CDR3 loop was 12.7 AA (range 9–18 AA) for thymus and 12.2 AA (range 5–18 AA) for peripheral blood, indicating that a reduced CDR3 length in the β chain is essential for αβ T cell function.

Altogether, these results suggest that a particularly long δ CDR3 may be a prerequisite to perform appropriate T cell functions in artiodactyl species and this is achieved by increasing the number of TRDD genes used in the rearrangement. This situation is quite different from humans and mice, confirming that differences between “γδ high” and “γδ low” species in distribution, diversity and function of γδ T cells may be substantial.

Furthermore, it should be noted that also in dromedary as in humans there are TRAV genes (TRAV33/DV6 genes), which are used for the production of δ chains. The two TRAV33/DV6 genes and the unique TRDV3 gene that our genomic analysis confirms to be positioned after the TRDC gene in inverted orientation, contribute strongly to the adult dromedary δ repertoire through hypermutation [5].

Finally, the genomic characterisation of the entire TRA/TRD region in *C. dromedarius* allowed us to roughly investigate the corresponding region in *C. bactrianus* and *C. ferus* highlighting how omics data can be transferred to more closely related evolutionarily species.

The genomic comparison stressed that the development of improved versions of genomic assemblies are needed to decipher complex genomic regions such as TRA/TRD genes. Moreover, high-quality genome assemblies serve not only as a reference to further genome assembly improvements, but also allow detailed studies of the diversity between genomes.

However, if high-quality genome assemblies are essential for the characterisation of complex genomic regions, detailed annotations of these regions are useful for validating the reliability of the assembly.

## 5. Conclusions

γδ T cells are an enigmatic cell population with unique features compared with αβ T cells. Their functional plasticity allows these T cells to be involved in adaptive and innate immune responses [11,47]. However, γδ T cells can be highly divergent between species and play specialised roles within a species [48]. In humans and mice, γδ T cells constitute a minority of the T cell pool (“γδ low species”). In addition, the γδ TR phenotypes generated by a somatic recombination are very limited and also the germline gene repertoire is very restricted.

In artiodactyls (sheep, cows, goats and pigs), γδ T cells represent an intriguing set of T population whose functions and characteristics have to be defined. However, in these species, γδ T cells exhibit a higher frequency and a wider physiological distribution with respect to “γδ low species” [13,14,15,16]. For this reason, ruminants and pigs are considered “γδ T cell high species”. Moreover, looking at the expressed γδ TR repertoire, the diversity is significantly higher in artiodactyl species than in humans and mice. This feature is favored by the large number of germline genes encoding for γ and δ chains, as proved by the genomic analysis of the TRG and TRD loci in the major livestock species (sheep, goat and cattle) [11,12,17].

Among artiodactyls, camels have also been defined “γδ high species” [4]. Previous expression studies on γ and δ chains have showed that also in dromedary, as in the other “γδ high species”, the primary γδ repertoire is wide and diversified, even if its diversity seems to be largely due to SHM rather than the number of the germline genes [5,6,7]. This assumption has been proved by analysing the genomic organisation of the dromedary TRG locus that revealed how the total number of TRG germline genes is certainly lower compared to that of sheep and cattle, and the γ chain diversity due to the potential gene rearrangements is therefore more limited.

The results of our study on the genomic organisation of the dromedary TRD locus definitively confirmed this finding, highlighting a lower number of germline genes available to generate δ chains compared with the other artiodactyls. Therefore, the observed diversity of dromedary γδ TR is higher than that expected due to the SHM effect. Furthermore, the incorporation up to four *D* genes greatly contribute to the diversity of the antigen binding-site of the receptor.

## Figures and Tables

**Table 1:** Description of the TRAV subgroups.

Subgroups	Gene Number	Functional	ORF/P
*TRAV1*	1	1	-
*TRAV2*	1	-	1
*TRAV3*	1	1	-
*TRAV4*	1	1	-
*TRAV8*	3	-	3
*TRAV9*	5	4	1
*TRAV12*	2	1	1
*TRAV13*	3	2	1
*TRAV14*	4	1	3
*TRAV16*	4	-	4
*TRAV17*	1	-	1
*TRAV18*	1	1	-
*TRAV19*	4	1	3
*TRAV22*	8	4	4
*TRAV23*	7	2	5
*TRAV24*	7	2	5
*TRAV25*	4	3	1
*TRAV26*	1	-	1
*TRAV27*	1	-	1
*TRAV28*	1	1	-
*TRAV29*	1	-	1
*TRAV31*	1	-	1
*TRAV33/DV6*	2	2	-
*TRAV35*	1	-	1
*TRAV36*	1	1	-
*TRAV37*	1	-	1
*TRAV38*	1	1	-
*TRAV39*	1	1	-
*TRAV40*	1	1	-
*TRAV41*	1	1	-
*TRAV43*	2	1	1
*TRAV44*	2	2	-
*TRAV45*	8	1	7
**Total**	**83**	**36**	**47**

**Table 2:** Correspondence between TRDV genes and nomenclatures.

This Work	Antonacci et al. [5]	% NN Identity
TRDV1-1_F	-	from 93.3 to 94.7 *
TRDV1-2_F	TRDV1-6	99.71
TRDV1-3_F	TRDV1-3	99.71
TRDV1-5_F	TRDV1-1	99.71
TRDV1-6_F	TRDV1-2	99.37
TRDV1-7_F	TRDV1-4	99.37
-	TRDV1-5	from 92.1 to 96.9 *
TRAV33-1/DV6-1	TRDV2.2	99.1
TRAV33-2/DV6-2	TRDV2.1	100
TRDV3	TRDV4	100

* indicates the identity range with all compared *TRDV1*.

**Table 3:** CDR3 length (AA) of the cDNAs.

		TRDV1	TRAV33/DV6	TRDV3	Total
Spleen	N° clones	9	7	6	22
	CDR3 length				
	mean value	22.8	21.1	20.3	21.6
	range	13–37	18–28	19–27	13–37
Tonsils	N° clones	8	4	3	15
	CDR3 length				
	mean value	21.2	19.2	13.3	19.1
	range	15–32	17–22	9–18	9–32
Blood	N° clones	22	-	2	24
	CDR3 length				
	mean value	20.4	-	18.5	20.3
	range	12–32	-	18–19	12–32
Total	N° clones	39	11	11	61
	CDR3 length				
	mean value	21.7	20.4	18.0	20.4
	range	12–37	17–28	9–27	9–37

**Table 4:** Correlation between the CDR3 length (AA) and the number of TRDD genes.

Subgroups		1 TRDD	2 TRDD	3 TRDD	4 TRDD
*TRDV1*	N° clones	5	11	13	6
	CDR3 length				
	mean value	19.6	18.3	21.6	28.3
	range	17–22	12–25	15–28	18–37
*TRAV33/DV6*	N° clones	2	6	3	-
	CDR3 length				
	mean value	19.0	21.3	19.6	-
	range	19	17–28	17–22	-
*TRDV3*	N° clones	7	3	1	-
	CDR3 length				
	mean value	17.8	18.3	19.0	-
	range	9–27	18–19	19	-
Total	N° clones	14	20	17	6
	CDR3 length				
	mean value	18.6	19.2	21.1	28.3
	range	9–27	12–28	15–28	18–37

## Data Availability

The data presented in this study are available within the article or supplementary material.

## Supplementary Materials

The following are available online at https://www.mdpi.com/article/10.3390/genes12040544/s1, Figure S1: Structure of the TRAV genes. The IMGT Protein display of the dromedary TRAV genes. The deduced amino acid sequences of the TRAV genes were manually aligned according to IMGT unique numbering for the V-REGION [35] to maximise homology. Only functional genes, ORF and in-frame pseudogenes are shown. All sequences exhibit the typical framework regions (FR) and complementarity determining regions (CDR) and the four amino acids (depicted and indicated in bold): cysteine 23 (1st-CYS) in FR1-IMGT, tryptophan 41 (CONSERVED-TRP) in FR2-IMGT, hydrophobic (here L, V and I) 89, and cysteine 104 (2nd-CYS) in FR3-IMGT, with the exception of TRAV23-2 and TRAV24-5 pseudogenes that lack the CONSERVED-TRP. Conversely, CDR-IMGT vary in amino acid composition and length. The description of the strands and loops and of the FR-IMGT and CDR-IMGT is according to the IMGT unique numbering for V-REGION [35]. The amino acid length of the CDR-IMGT is also indicated in square brackets. Figure S2: The IMGT Protein display of the dromedary TRDV genes. The deduced amino acid sequences of the TRDV genes were manually aligned according to IMGT unique numbering for the V-REGION [35] to maximise homology. Only functional genes are shown. All sequences exhibit the typical framework regions (FR) and complementarity determining regions (CDR) and the four amino acids (depicted and indicated in bold): cysteine 23 (1st-CYS) in FR1-IMGT, tryptophan 41 (CONSERVED-TRP) in FR2-IMGT, hydrophobic (here L) 89, and cysteine 104 (2nd-CYS) in FR3-IMGT. Conversely, CDR-IMGT vary in amino acid composition and length. The description of the strands and loops and of the FR-IMGT and CDR-IMGT is according to the IMGT unique numbering for V-REGION [35]. The amino acid length of the CDR-IMGT is also indicated in square brackets. Figure S3: Zoom of the schematic representation of Figure 1 for the genomic organisation of the dromedary TRD D-J-C and TRA J-C clusters as deduced from the genome assembly CamDro3. The diagram shows the position of all TRA and TRD genes according to nomenclature. The boxes representing the genes are not to scale. The exons are not shown. The arrows indicate the transcriptional orientation of the genes. Figure S4: Nucleotide and deduced amino acid sequences of the dromedary TRDD (A), TRDJ (B) and TRDC (C) genes. The consensus sequence of the heptamer and nonamer is provided at the top of the figure and is underlined. The name adopted for the gene classification is reported on the left of each gene. In (A), the inferred amino acid sequence of the TRDD genes in the three coding frames are reported. In (B), the donor splice site for each TRDJ is shown. The canonical F-G-X-G amino acid motifs are underlined. In (C), IMGT Protein display of the dromedary TRDC gene. The descriptions of the strands and loops were collected according to the IMGT unique numbering for the C-DOMAIN [49]. The abbreviation for the connecting (CO), transmembrane (TM) and cytoplasmic (CY) regions is used. Figure S5: The NJ tree inferred from the dromedary, sheep and human TRDJ gene sequences. The orthologous genes of the three species form monophyletic groups. The evolutionary analyses were conducted in MEGA X [30]. The optimal tree with the sum of branch length = 1.64240724 is shown. The percentage of replicate trees in which the associated taxa clustered together in the bootstrap test (100 replicates) are shown next to the branches [36]. The tree is drawn to scale, with branch lengths in the same units as those of the evolutionary distances used to infer the phylogenetic tree. The evolutionary distances were computed using the p-distance method [33] and are in the units of the number of base differences per site. This analysis involved 12 nucleotide sequences. Codon positions included were 1st + 2nd + 3rd + Noncoding. All ambiguous positions were removed for each sequence pair (pairwise deletion option). There was a total of 94 positions in the final dataset. The IMGT 6-letter for species (Camdro, Oviari and Homsap) standardized abbreviation for taxon is used. Figure S6: Nucleotide and deduced amino acid sequences of the dromedary TRAJ (A) and TRAC (B) genes. In (A), nucleotide and deduced AA sequences of the dromedary TRAJ genes. The numbering adopted for the gene classification is reported on the left of each gene. The functionality is also reported. The sequences of the J-heptamer and J-nonamer are well conserved for all TRAJ, except for the TRAJ19 and TRAJ55 genes, with respect to the consensus sequences provided at the top of the figure and underlined. The donor splice site for each TRAJ is also shown. The canonical F/W-G-X-G amino acid motifs are underlined. In (B), IMGT Protein display of the TRAC gene. Description of the strands and loops is according to the IMGT unique numbering for C-DOMAIN [49]. The abbreviation for the connecting (CO), transmembrane (TM) and cytoplasmic (CY) regions is used. Figure S7: CDR3 nucleotide and predicted amino acid sequences retrieved from the TRD cDNA clones isolated from spleen, tonsils and peripheral blood. CDR3-IMGT sequences are shown from codon 105 (the codon after the 2nd-CYS 104 of the V-REGION) to codon 117 (the codon before J-PHE 118 of the J-REGION) according to the unique numbering [35]. The CDR3 nucleotide/amino acid sequence, and the classification of the TRDV and TRDJ genes of each clone are also listed. The TRDV genes were assigned to the corresponding germline subgroup only. Nucleotides of the 3′V-REGION and of the 5′J-REGION are indicated in uppercase letters. The sequences considered to present recognizable TRDD genes are indicated in colored lowercase letters (pink for TRDD1, blue for TRDD2, red for TRDD3, orange for TRDD5, green for TRDD6 and dark purple for the TRDD missing in the current assembly), and the nucleotide substitutions are underlined. The amino acids belonging to a TRDD gene are in bold. Nucleotides that cannot be attributed to any V, D or J region (P/N-nucleotides), are indicated in lower cases on the left and on the right sides of the TRDD regions. The CDR3 length (AA and NN), with the contribution of TRDV, TRDD, TRDJ and P/N nucleotide sequences, and the name of each clone are also reported. Figure S8: Graphic representation of the ratio between the length (mean values) of P/N and TRDD nucleotide sequences in the cDNAs groups according to the number of TRDD genes. Each cDNA group consists of clones derived from spleen, tonsils and blood. The TRDD and N/P length values were retrieved from Supplementary Figure S7. Table S1: Description of the TRA/TRD genes in the *C. dromedarius* chromosome 6 genome assembly (GeneBank ID: NC_044516.1). The position of all genes and their classification and functionality are reported.

## References

[1] Russell J.B., Wilson D.B. (1996). Why are ruminal cellulolytic bacteria unable to digest cellulose at low pH?. J. Dairy Sci..

[2] Dehority B.A. (2002). Gastrointestinal tracts of herbivores, particularly the ruminant: Anatomy, physiology and microbial digestion of plants. J. Appl. Anim. Res..

[3] Ciccarese S., Burger P.A., Ciani E., Castelli V., Linguiti G., Plasil M., Massari S., Horin P., Antonacci R. (2019). The Camel Adaptive Immune Receptors Repertoire as a Singular Example of Structural and Functional Genomics. Front. Genet..

[4] Hussen J., Schuberth H.J. (2021). Recent Advances in Camel Immunology. Front. Immunol..

[5] Antonacci R., Mineccia M., Lefranc M.-P., Ashmaoui H.M., Lanave C., Piccinni B., Pesole G., Hassanane M.S., Massari S., Ciccarese S. (2011). Expression and genomic analyses of *Camelus dromedarius* T cell receptor delta (TRD) genes reveal a variable domain repertoire enlargement due to CDR3 diversification and somatic mutation. Mol. Immunol..

[6] Vaccarelli G., Antonacci R., Tasco G., Yang F., El Ashmaoui H.M., Hassanane M.S., Massari S., Casadio R., Ciccarese S. (2012). Generation of diversity by somatic mutation in the *Camelus dromedarius* T-cell receptor gamma (TCRG) variable domains. Eur. J. Immunol..

[7] Ciccarese S., Vaccarelli G., Lefranc M.-P., Tasco G., Consiglio A., Casadio R., Linguiti G., Antonacci R. (2014). Characteristics of the somatic hypermutation in the *Camelus dromedarius* T cell receptor gamma (TRG) and delta (TRD) variable domains. Dev. Comp. Immunol..

[8] Lefranc M.-P. (2014). Immunoglobulin and T Cell Receptor Genes: IMGT^®^ and the Birth and Rise of Immunoinformatics. Front. Immunol..

[9] Lefranc M.-P., Lefranc G. (2019). IMGT^®^ and 30 Years of Immunoinformatics Insight in Antibodg V and C Domain Strucrure and Function. Antibodies.

[10] Antonacci R., Linguiti G., Burger P.A., Castelli V., Pala A., Fitak R., Massari S., Ciccarese S. (2020). Comprehensive genomic analysis of the dromedary T cell receptor gamma (TRG) locus and identification of a functional TRGC5 cassette. Dev. Comp. Immunol..

[11] Antonacci R., Massari S., Linguiti G., Caputi Jambrenghi A., Giannico F., Lefranc M.P., Ciccarese S. (2020). Evolution of the T-Cell Receptor (TR) Loci in the Adaptive Immune Response: The Tale of the TRG Locus in Mammals. Genes.

[12] Giannico F., Massari S., Caputi Jambrenghi A., Soriano A., Pala A., Linguiti G., Ciccarese S., Antonacci R. (2020). The expansion of the TRB and TRG genes in domestic goats (Capra hircus) is characteristic of the ruminant species. BMC Genom..

[13] Hein W.R., Dudler L. (1993). Divergent evolution of T-cell repertoire: Extensive diversity and developmentally regulated expression of the sheep γδ T-cell receptor. EMBO J..

[14] Hein W.R., Dudler L. (1997). TCR cells are prominent in normal bovine skin and express a diverse repertoire of antigen receptors. Immunology.

[15] Yang Y.G., Ohta S., Yamada S., Shimizu M., Takagaki Y. (1995). Diversity of T cell receptor delta-chain cDNA in the thymus of a one-month-old pig. J. Immunol..

[16] Antonacci R., Lanave C., Del Faro L., Vaccarelli G., Ciccarese S., Massari S. (2005). Artiodactyl emergence is accompanied by the birth of an extensive pool of diverse germline TRDV1 genes. Immunogenetics.

[17] Pégorier P., Bertignac M., Nguefack Ngoune V., Folch G., Jabado-Michaloud J., Giudicelli V., Duroux P., Lefranc M.P., Kossida S. (2020). IMGT^®^ Biocuration and Comparative Analysis of *Bos taurus* and *Ovis aries* TRA/TRD Loci. Genes.

[18] Lado S., Elbers J.P., Rogers M.F., Melo-Ferreira J., Yadamsuren A., Corander J., Horin P., Burger P.A. (2020). Nucleotide diversity of functionally different groups of immune response genes in Old World camels based on newly annotated and reference-guided assemblies. BMC Genom..

[19] Schwartz S., Zhang Z., Frazer K.A., Smit A., Riemer C., Bouck J., Gibbs R., Hardison R., Miller W. (2000). PipMaker—A web server for aligning two genomic DNA sequences. Genome Res..

[20] Ming L., Wang Z., Yi L., Batmunkh M., Liu T., Siren D., He J., Juramt N., Jambl T., Li Y. (2020). Chromosome-level assembly of wild Bactrian camel genome reveals organization of immune gene loci. Mol. Ecol. Resour..

[21] Lefranc M.-P., Forster A., Baer R., Stinson M.A., Rabbitts T.H. (1986). Diversity and rearrangement of the human T cell rearranging gamma genes: Nine germ-line variable genes belonging to two subgroups. Cell.

[22] Brochet X., Lefranc M.-P., Giudicelli V. (2008). IMGT/V-QUEST: The highly customized and integrated system for IG and TR standardized V-J and V-D-J sequence analysis. Nucleic Acids Res..

[23] Giudicelli V., Chaume D., Lefranc M.-P. (2005). IMGT/GENE-DB: Comprehensive database for human and mouse immunoglobulin and T cell receptor genes. Nucleic Acids Res..

[24] Lefranc M.-P., Giudicelli V., Duroux P., Jabado-Michaloud J., Folch G., Aouinti S., Carillon E., Duvergey H., Houles A., Payson-Lafosse T. (2015). IMGT^®^, the international ImMunoGenetics information system^®^ 25 years on. Nucleic Acids Res..

[25] Lefranc M.-P. (2011). From IMGT-ONTOLOGY CLASSIFICATION Axiom to IMGT standardized gene and allele nomenclature: For immunoglobulins (IG) and T cell receptors (TR). Cold Spring Harb. Protoc..

[26] Lefranc M.-P. (2001). Nomenclature of the human T cell receptor genes. Curr. Protoc. Immunol..

[27] Lefranc M.-P., Lefranc G. (2001). The T Cell Receptor FactsBook.

[28] Scaviner D., Lefranc M.-P. (2000). The human T cell receptor alpha variable (TRAV) genes. Exp. Clin. Immunogenet..

[29] Edgar R.C. (2004). MUSCLE: A multiple sequence alignment with reduced time and space complexity. BMC Bioinform..

[30] Kumar S., Stecher G., Li M., Knyaz C., Tamura K. (2018). MEGA X: Molecular Evolutionary Genetics Analysis across computing platforms. Mol. Biol. Evol..

[31] Stecher G., Tamura K., Kumar S. (2020). Molecular Evolutionary Genetics Analysis (MEGA) for macOS. Mol. Biol. Evol..

[32] Saitou N., Nei M. (1987). The neighbor-joining method: A new method for reconstructing phylogenetic trees. Mol. Biol. Evol..

[33] Nei M., Kumar S. (2000). Molecular Evolution and Phylogenetics.

[34] Piccinni B., Massari S., Caputi Jambrenghi A., Giannico F., Lefranc M.-P., Ciccarese S., Antonacci R. (2015). Sheep (*Ovis aries*) T cell receptor alpha (TRA) and delta (TRD) genes and genomic organization of the TRA/TRD locus. BMC Genom..

[35] Lefranc M.-P., Pommié C., Ruiz M., Giudicelli V., Foulquier E., Truong L., Thouvenin-Contet V., Lefranc G. (2003). IMGT unique numbering for immunoglobulin and T cell receptor variable domains and Ig superfamily V-like domains. Dev. Comp. Immunol..

[36] Felsenstein J. (1985). Confidence limits on phylogenies: An approach using the bootstrap. Evolution.

[37] Lefranc M.-P. (2011). IMGT Collier de Perles for the Variable (V), Constant (C), and Groove (G) Domains of IG, TR, MH, IgSF, and MhSF. Cold Spring Harb. Protoc..

[38] Herzig C.T., Lefranc M.-P., Baldwin C.L. (2010). Annotation and classification of the bovine T cell receptor delta genes. BMC Genom..

[39] Uenishi H., Eguchi-Ogawa T., Toki D., Morozumi T., Tanaka-Matsuda M., Shinkai H., Yamamoto R., Takagaki Y. (2009). Genomic sequence encoding diversity segments of the pig TCR delta chain gene demonstrates productivity of highly diversified repertoire. Mol. Immunol..

[40] Antonacci R., Bellini M., Ciccarese S., Massari S. (2019). Comparative analysis of the TRB locus in the *Camelus* genus. Front. Genet..

[41] Fichtner A.S., Karunakaran M.M., Gu S., Boughter C.T., Borowska M.T., Starick L., Nöhren A., Göbel T.W., Adams E.J., Herrmann T. (2020). Alpaca (*Vicugna pacos*), the first nonprimate species with a phosphoantigen-reactive Vγ9Vδ2 T cell subset. Proc. Natl. Acad. Sci. USA.

[42] Shin S., El-Diwany R., Schaffert S., Adams E.J., Garcia K.C., Pereira P., Chien Y.H. (2005). Antigen recognition determinants of gammadelta T cell receptors. Science.

[43] Vermijlen D., Gatti D., Kouzeli A., Rus T., Eberl M. (2018). γδ T cell responses: How many ligands will it take till we know?. Semin. Cell Dev. Biol..

[44] Antonacci R., Bellini M., Pala A., Mineccia M., Hassanane M.S., Ciccarese S., Massari S. (2017). The occurrence of three D-J-C clusters within the dromedary TRB locus highlights a shared evolution in Tylopoda, Ruminantia and Suina. Dev. Comp. Immunol..

[45] Di Tommaso S., Antonacci R., Ciccarese S., Massari S. (2010). Extensive analysis of D-J-C arrangements allows the identification of different mechanisms enhancing the diversity in sheep T cell receptor beta-chain repertoire. BMC Genom..

[46] Massari S., Bellini M., Ciccarese S., Antonacci R. (2018). Overview of the Germline and Expressed Repertoires of the TRB Genes in *Sus scrofa*. Front. Immunol..

[47] Papadopoulou M., Sanchez Sanchez G., Vermijlen D. (2020). Innate and adaptive γδ T cells: How, when, and why. Immunol. Rev..

[48] Herrmann T., Karunakaran M.M., Fichtner A.S. (2020). A glance over the fence: Using phylogeny and species comparison for a better understanding of antigen recognition by human γδ T-cells. Immunol. Rev..

[49] Lefranc M.-P., Pommié C., Kaas Q., Duprat E., Bosc N., Guiraudou D., Jean C., Ruiz M., Da Piédade I., Rouard M. (2005). IMGT unique numbering for immunoglobulin and T cell receptor constant domains and Ig superfamily C-like domains. Dev. Comp. Immunol..

