# Supplementary figures and images for: The Genomic Organisation of the TRA/TRD Locus Validates the Peculiar Characteristics of Dromedary δ-Chain Expression
Source: Genes (Basel). 2021 Apr 9;12(4):544. doi: 10.3390/genes12040544 (PMC8069558; doi:10.3390/genes12040544)

## RATIO P-N/TRDD

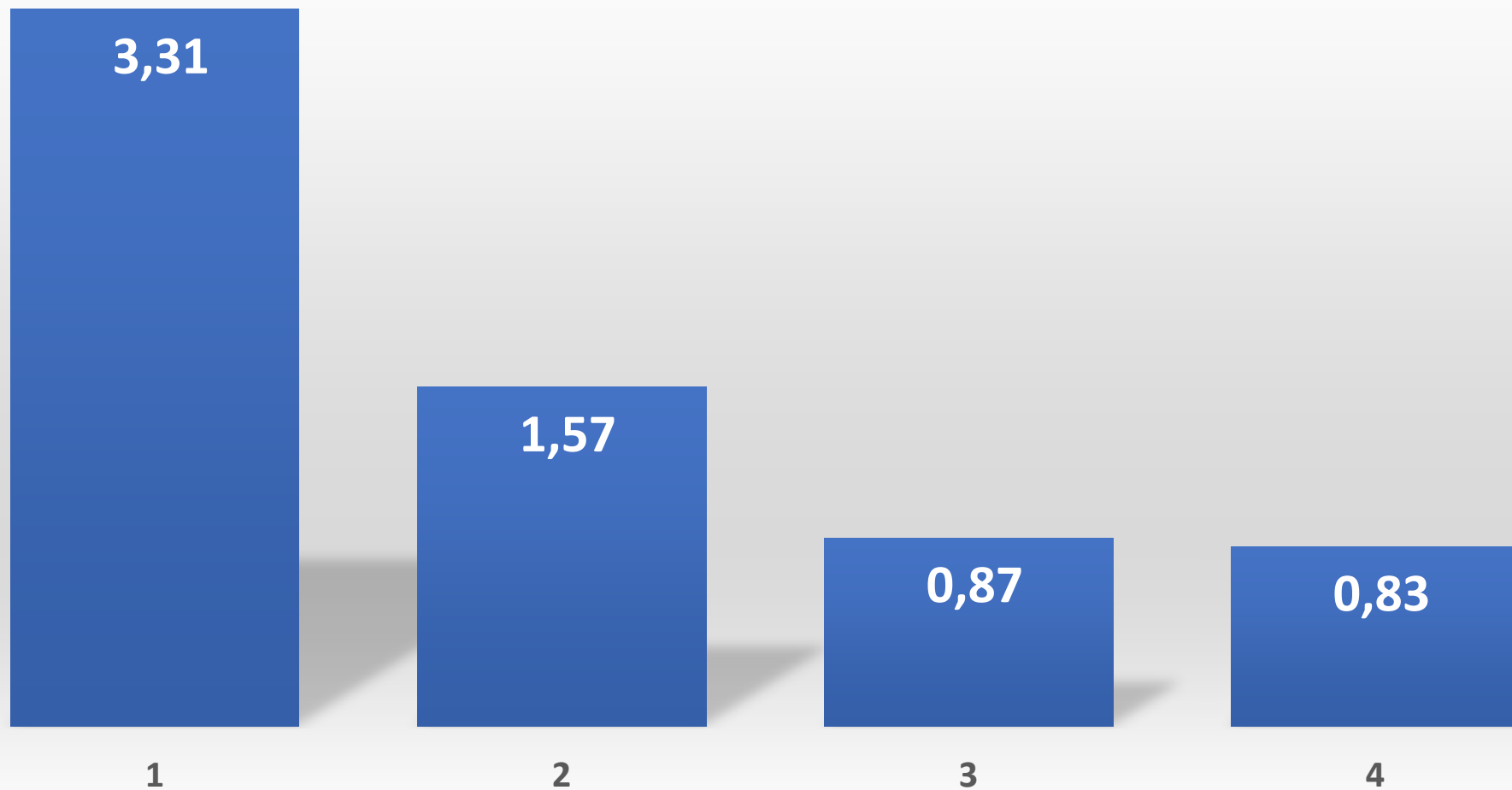

TRDD genes

Supplement: Supplementary file 1 [file genes-12-00544-s001.zip › Supplementary files /Supplementary Figure S8.pdf]

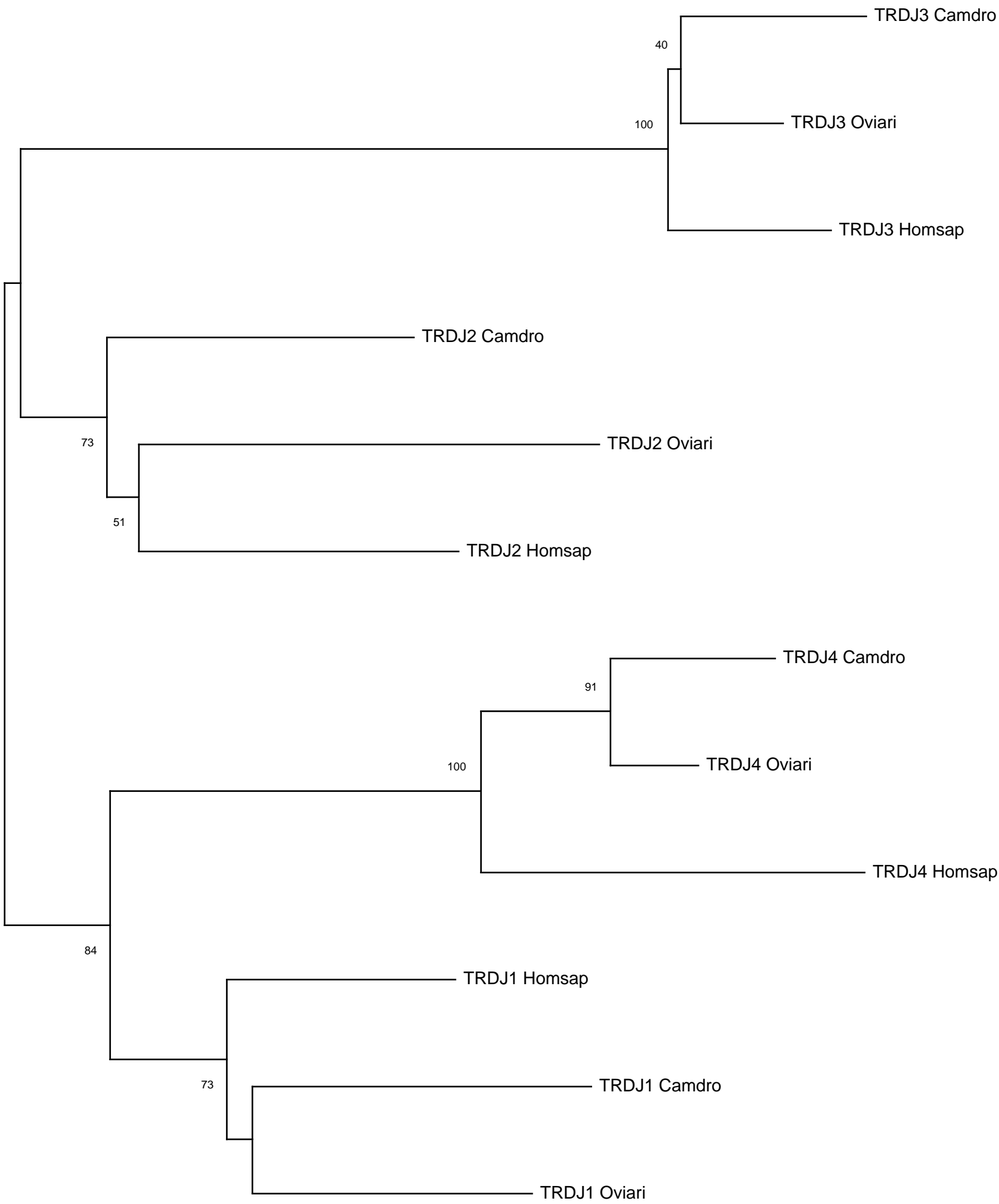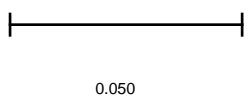

Supplement: Supplementary file 1 [file genes-12-00544-s001.zip › Supplementary files /Supplementary Figure S5.pdf]
