# Supplementary material for: The Genomic Organisation of the TRA/TRD Locus Validates the Peculiar Characteristics of Dromedary δ-Chain Expression
Source: Genes (Basel). 2021 Apr 9;12(4):544. doi: 10.3390/genes12040544 (PMC8069558; doi:10.3390/genes12040544)
Supplement: Supplementary file 1 [file genes-12-00544-s001.zip › Supplementary files /Supplementary Figure S6.doc]

**(A)**

| **Gene name** | **Functionality** | **J-NONAMER**  **GGTTTTTGT** | **12–spacer** | **J-HEPTAMER**  **CACTGTG** | **TRAJ** | **5'splice donor** |
| --- | --- | --- | --- | --- | --- | --- |
| TRAJ1 | F | gctttctgt | aatggagacatt | gggagtg | GTATGGAGGTGTTGCCTCCCAGCTGCAATTTGGCAAGGGAACCAGAGTTTCCATTACTCCTG  Y G G V A S Q L Q F G K G T R V S I T P | gtaagt |
| TRAJ2 | F | agtttctgt | aatggggtcacc | tgctgtg | TGAATACTGGAGGAACGGCTGGTAAACTCACATTTGGGAAAGGAACCCAAGTATCCATAATATCTG  N T G G T A G K L T F G K G T Q V S I I S | gtgagt |
| TRAJ3 | F | ggttattgc | aaagaccttccc | ctcagtg | GGGATTCAACAGTGGTAACAAGTTAATCTTTGGAGCAGGGACCAGACTGAGTGTCCAGCCAA  G F N S G N K L I F G A G T R L S V Q P | gtaagg |
| TRAJ4 | F | ttcttgtaa | agcatcctccta | gggtgtg | CTGTCTGGTAGCTACAGTAAGCTGATCTTTGGAGCCGGGACCAGGCTGGCTGTACGCCCAT  L S G S Y S K L I F G A G T R L A V R P | gtgagt |
| TRAJ5 | F | agtatttgt | ataacgttgcac | cagggtg | CGAACACAGGCAGCAGAACACTCACTTTTGGGAGAGGAACCAGACTCCAAGTGCATCCAA  N T G S R T L T F G R G T R L Q V H P | gtaagt |
| TRAJ6 | F | ggttttggc | aaaggctttccc | tgctgtg | TGTATCAGGAGGAAACTATGGACTTACATTTGGAAGAGGGACCAGGCTTGTTGTTCATCCAT  V S G G N Y G L T F G R G T R L V V H P | gtgagt |
| TRAJ7 | F | cctttttgt | aatgcactgacc | tggagtg | GGATTATGAAAACAGATTCACTTTTGGGAAGGGGACCCAAGTAGTGGTCACACCAA  D Y E N R F T F G K G T Q V V V T P | gtaagt |
| TRAJ8 | F | attttgtat | agaggtacatca | gagtgtg | AACACAGACTACCAGAAGCTCACATTTGGAATTGGCACCCAACTTTTGATCAGCCCAA  N T D Y Q K L T F G I G T Q L L I S P | gtaagt |
| TRAJ9 | F | ccattttgt | cacagcacacat | cactgtg | GGCAATACTGGAGCCTTCAAATTTGTCTTTGGGACAGGAACAAGACTATTTGTTGAAGCAA  G N T G A F K F V F G T G T R L F V E A | gtaagt |
| TRAJ10 | F | agtttactg | tgaggcatcacg | cactgtg | GGACTCACGGGAGGAGCAAACAAGCTCACCTTCGGGAAAGGCACCCGGCTAAAAGTGGAACTGA  G L T G G A N K L T F G K G T R L K V E L | gtaagt |
| TRAJ11 | F | cctttttgt | atgggggtttgc | tagagtg | TGAACTCAGGATACAACACACTTACCTTTGGAAAGGGGACTGTGCTTCTGGTCTCTCCAG  N S G Y N T L T F G K G T V L L V S P | gtaaat |
| TRAJ12 | F | ggttctggg | ctgactgtgaac | cactgtg | GGAGTGATGGAGGCTACAGATGGACCTTCGGGAGTGGGACCAGACTGCTGGTCAGGCCTG  S D G G Y R W T F G S G T R L L V R P | gtaagt |
| TRAJ13 | F | attctgtaa | aggcctgcattg | tggtgtg | AATTTTGGGGGCAGCCAAAAGGTTACCTTTGGAACTGGAACAATGCTTCGGGTCACCCCAA  N F G G S Q K V T F G T G T M L R V T P | gtaagt |
| TRAJ14 | ORF(1) | aattgttgt | caggtagcgcgg | tgctgtg | ATTTATAACACATTCATCTTTGGGAGAGGGACAAGATTATCAGTAAAACCCA  I Y N T F I F G R G T R L S V K P | gcaagc |
| TRAJ15 | F | ggtattcgc | agggcctcattt | cactgtg | CCTACCAGGGAGGAAGTGCGCTGACCTTTGGGAAAGGAACCATCGTATCAGTGAGTCCCA  Y Q G G S A L T F G K G T I V S V S P | gtaagt |
| TRAJ16 | F | ggtttttgt | ggtgaaatagat | cactgtg | GGGTTTTCAAGCACCCAGAAGCTGGTATTTGGAAGGGGGACCATGGTGCAGGTGAATCTTA  G F S S T Q K L V F G R G T M V Q V N L | gtaagt |
| TRAJ17 | F | ggtttttgt | tgggcctcaaat | cattgtg | TGACCAACACTGCAGGAAACAAGCTAATCTTTGGAGGAGGAACCAGGGTGTCAGTCAAGCCGA  T N T A G N K L I F G G G T R V S V K P | gtgagt |
| TRAJ18 | F | ggttcatgc | aaaggggcccag | cactgtg | TCGACAGAGGCTCAAGTCTGGGGAAGCTGTACTTTGGAAGAGGAACTCAGCTAATCGTATGGCCTG  D R G S S L G K L Y F G R G T Q L I V W P | gtgagt |
| TRAJ19 | P(2) | ggtgatttt | gcagaggacaga | tctagtc | TATCAGAATTTTCACAAGTTCAGCTTTGGAAAGGGATCCGAGTAATGACAATCCAA  Y Q N F H K F S F G K G S E * * Q S | gtaagt |
| TRAJ20 | F | cgtttgtgt | aggaaggcgtag | cactgtg | GCTCTAATGACTACAAGCTCACCTTTGGAGCAGGAACCACGGTAACTGTAAGAGCAA  S N D Y K L T F G A G T T V T V R A | gtaagt |
| TRAJ21 | F | gctttttgt | aatgctagtaaa | cacagtg | TACAACACCAACAGACTGTACTTCGGCTCTGGGACCAAACTCAGTGTAAAGCCAA  Y N T N R L Y F G S G T K L S V K P | gtaagt |
| TRAJ22 | F | ggtttttgt | tgttgggcacat | cacagtg | TTTCTTCTGGTTCCGGCTGGCAACTGACCTTTGGATCTGGGACCCAGCTGACGGTTGTACCTG  S S G S G W Q L T F G S G T Q L T V V P | gtaggc |
| TRAJ23 | F | tgtttttga | caggctgtgtag | cactgtg | TGAATTATAACCAGGGAGGAAAGCTTATCTTCGGACAGGGAACCGAGTTATCTGTGAAGCCCA  N Y N Q G G K L I F G Q G T E L S V K P | gtaagt |
| TRAJ24 | F | ctattttgt | agaggtgtttgt | cacagtg | TGACAACTGACGGCTGGGGGAAATTGAATTTTGGAGCAGGGACCCGGATTGTGGTCACCCCAG  T T D G W G K L N F G A G T R I V V T P | gtaagc |
| TRAJ25 | F | ggtttttga | tgctgagataat | cactgtg | TGGGAGGACAAGGCTTCTCCCTCACCTTTGGGAAGGGGACGAGGCTGCTTGTTAAGCCGA  G G Q G F S L T F G K G T R L L V K P | gtaagt |
| TRAJ26 | F | cgtttttgc | agagcccggagg | cactgtg | GGAATAACTACGCCCAGGGTCTGACCTTCGGTGGGGGAACCAGATTGTCGGTGCTGCCCT  N N Y A Q G L T F G G G T R L S V L P | gtaagt |
| TRAJ27 | F | ggttattac | aatagcactgaa | cactgtg | TGACACCAGTGCAGGCAGATTAACCTTTGGGAACGGGACCGCGCTCACTGTGAAGCCAA  D T S A G R L T F G N G T A L T V K P | gtaagt |
| TRAJ28 | F | ggtttttgc | aaagaaaggaaa | ctgtgtg | TGTACTCTGGAGCTGGGGGTTACCAACTCACTTTTGGGAAGGGCACCAAACTCCTGGTCACACCAA  Y S G A G G Y Q L T F G K G T K L L V T P | gtaagt |
| TRAJ29 | F | agtttctgt | tatggagacaat | cactgtg | GGGATTCAGGAGGGCGGCAGCTGGTCTTCGGAAAGGGCACAAGACTTTCTGTGATTCCAA  D S G G R Q L V F G K G T R L S V I P | gtaagt |
| TRAJ30 | F(3) | gttttttgt | cttagcctcaat | cacggtg | TGACAAACTTCCGGCACAACTGTCTTTGGAAAAGGGACTCAGCTGCACGTTCTCCCCA  * Q T S G T T V F G K G T Q L H V L P | gtaagt |
| TRAJ31 | F | ggtttcagt | aaaggcaggaaa | tgctgtg | GGAATAATAATGCCAGAATCTTCTTTGGAACTGGAACCCAGGTGGTGGTAAAGCCCA  N N N A R I F F G T G T Q V V V K P | gtaagt |
| TRAJ32 | F | ggttattgt | aaggctctgaag | ggctgtg | TAAATTACGGGGGTGCTGGAAACCAGCTCATCTTTGGGACCGGCACTCTGCTCTCTGTCAAGCCAA  N Y G G A G N Q L I F G T G T L L S V K P | gtacgt |
| TRAJ33 | F | agtttttgt | taaggtttttgt | ctctgtg | TGGATAGCAACTATCAGTTGACCTGGGGCTCTGGGACCAAGCTAATTATAAAGCCAG  D S N Y Q L T W G S G T K L I I K P | gtaagt |
| TRAJ34 | F | ggtttttgt | agacctcagtat | cactgtg | TCCTACAACAGTGACAAACTCATCTTTGGGGCTGGAACCAGATTACACGTCTTTCCAA  S Y N S D K L I F G A G T R L H V F P | gtaagt |
| TRAJ35 | F | ggtttttgt | agtggagtcagc | tactgtg | GGCAGACAGGCTCTGGGGCTGCGCTGCATTTCGGGTCCGGCACTCAAGTGATTGTTACACCAC  Q T G S G A A L H F G S G T Q V I V T P | gtaagt |
| TRAJ36 | F | ggattttgt | actgggcagaaa | cactgtg | CCAACTTCCGGGGGAAACCGGCTTGTCTTTGGGACTGGAACAGGACTCACCGTCATTCCCC  P T S G G N R L V F G T G T G L T V I P | gtaagt |
| TRAJ37 | F | agtttttgt | aaagggcagcat | tacagtg | TGGCTCTAGTAACACAGGAAAACTCGCCTTTGGGCAGGGAACCATGTTACAAGTAAAACCAG  G S S N T G K L A F G Q G T M L Q V K P | gtaggt |
| TRAJ38 | F | actttccgt | aaagctttctat | gactgtg | TAATATTGGCAACAACCGTAAGCTGATTTGGGGATTGGGGACAAGCCTGGCAGTAAATCCAA  N I G N N R K L I W G L G T S L A V N P | gtaagt |
| TRAJ39 | F | ggtttttgc | tgagctgaagag | cactgtg | TGAATAATAATGCAGGCAGCACGTTCACGTTTGGAGGGGGAACAAGGTTAATGGTCAAACCCT  N N N A G S T F T F G G G T R L M V K P | gtgagt |
| TRAJ40 | F | ggtttatgt | agagccacagag | cacggtg | ACTCAGGAAACTACAAATATACCTTTGGAGCAGGCACTAGCTTGCAGGTTTTAACAA  S G N Y K Y T F G A G T S L Q V L T | gtgagt |
| TRAJ41 | F | tgtttttgt | ttagggaaaatg | cactgtg | AACTCAGGCACGGGGTATGCACTCAACTTTGGCAAAGGCACCTCGCTGCTGGTCACACCTG  N S G T G Y A L N F G K G T S L L V T P | gtgagc |
| TRAJ42 | F | gattattgt | aaggccccatat | gactgtg | TGAATTATGGAGGCAGCCAAGGAAAGCTCATCTTTGGAAAAGGCACTGGACTTTCTGTTAAACCAA  N Y G G S Q G K L I F G K G T G L S V K P | gtaagt |
| TRAJ43 | F | ggtttttgt | tagagggtgtat | tgctgtg | GCAATAATAACAACGCCCCCCGCTTTGGAGCAGGAACCAGACTGACAGTAAAACCAA  N N N N A P R F G A G T R L T V K P | gtaagt |
| TRAJ44 | F | cgtttctgt | cgtggagtgtct | cacagtg | TCACCACTGGCAGTGGCAGGAAACTCATCTTTGGGACTGGAACAAGACTTCGGGTCACCGTTG  T T G S G R K L I F G T G T R L R V T V | gtaggt |
| TRAJ45 | F | gatttatgt | gaagggttgact | cagggtg | TGAATACAGGAGGAGTTACTGACAGACTCGTCTTTGGGAAAGGGACCCAGCTCACCATCCAGCCCT  N T G G V T D R L V F G K G T Q L T I Q P | gtaagt |
| TRAJ46 | F | tgtttctgt | aaagctgctgag | gacagtg | AGAAGAGAGGCAGCGGAGACAAGCTGACTTTTGGGACCGGGACGCGTTTAGCAGTGAGGCCCA  K R G S G D K L T F G T G T R L A V R P | gtaagt |
| TRAJ47 | F | tgtttttgt | agaggagtctga | tgctgtg | TGAATTATGGAAACACGCTAACGTTTGGCTCGGGAACCCTCTTGAGAGTCAGGCCTC  N Y G N T L T F G S G T L L R V R P | gtgagt |
| TRAJ48 | F | tttttgtta | agacttaaaaca | cagtgtg | TCTAACTACGGAAACAACAAATTTACCTTCGGGACAGGAACGAGGCTCACCGTTACACCCA  S N Y G N N K F T F G T G T R L T V T P | gtaagt |
| TRAJ49 | F | ggtttttgt | tgagcttcctat | cacagtg | TTAACACTGGCAACCAGAACTTCTATTTTGGGAGAGGGACGCGTTTGACAGTCGTTCCAA  N T G N Q N F Y F G R G T R L T V V P | gtaagt |
| TRAJ50 | F | agtttttgc | aaagggttgggt | ggctgtg | TGACAACTACAACAAGCTGACGTTTGGGCGGGGAACGAGCTTATCCGTCACTCCAA  D N Y N K L T F G R G T S L S V T P | gtaagt |
| TRAJ52 | F | ggttcttgt | aaaggcctccac | tgcagtg | CTAACACTGGTGGTGGTGGCTATGGAAAGCTAACATTTGGACAAGGGACCATCCTGACTATCCGCCCAA  N T G G G G Y G K L T F G Q G T I L T I R P | gtaagt |
| TRAJ53 | F | tgtttttgt | aaagcctcccct | gactgtg | AGAATAGTGGAGGTAGCAGCTATAAACTGACATTTGGAAAAGGAACTCTCTTGATTGTGAATCCAA  N S G G S S Y K L T F G K G T L L I V N P | gtaagt |
| TRAJ54 | F | ccaggtttg | tgtaaagctatt | tgctgtg | GTGCAGCCCAGCCAGGAGCTGGTATTCGGGCAAGGAACCAGGCTGACCATCATCCCCA  V Q P S Q E L V F G Q G T R L T I I P | gtgagt |
| TRAJ55 | P(4) | aagctttgc | ctgtcagagaga | atctaag | AGCTGAAAAGATGGGAAGCTGACATTTGGAAACTAGATTAAAAAGAAAATTCAAACGAATCCC  S * K D G K L T F G N * I K K K I Q T N P | gaggag |
| TRAJ56 | F | gttttttgt | agagtcgcgtgt | cattgtg | TGGTATGGCACCGACAATAAGCTGATATTTGGAAAAGGAACAACTCTGAGTGTTCGGCCAG  W Y G T D N K L I F G K G T T L S V R P | gtatgt |
| TRAJ57 | F | ggtatttgt | aaagcagtctga | gggggtg | TAACTCAGGGTGGAGCCGAAAGAATCTTCTTTGGAAAGGGAACGAAACTGACAGTGAGCCCAT  T Q G G A E R I F F G K G T K L T V S P | gtaagt |
| TRAJ58 | F | ggtttttgc | aaagccccttgg | cacagtg | TTCAGCAAGCCAGTGGCACTAAGATGACGTTTGGGAAGGGGACTCAGCTCACAGTGAATCTTG  Q Q A S G T K M T F G K G T Q L T V N L | gtaagt |
| TRAJ59 | ORF(5) | gtattatgt | aaaggcaacagc | tcctgtg | GGAAGGACGGAAATAGGAAATTTACATTTGGAACCAGGGCTCAAGTGAGAGTGAAACTTA  K D G N R K F T F G T R A Q V R V K L | gtaagc |
| TRAJ60 | F | acttttggt | aaagggtggagg | caccgtg | TGAAGGGCACTGAGAAGCCCGCCTTCGGGAAGGGGACTCAGTTAATCGTAAGCCTGG  K G T E K P A F G K G T Q L I V S L | gtgagt |
| TRAJ61 | P(6) | ggtttttgt | aaaggtgttcac | tcctgtg | GGAATGGGGTTAATAGGAAACTGACATTTGGAGCCAAAACTTGATACTCA  N G V N R K L T F G A K T * Y S | gtaagt |

```
Notes:
(1)defective splice site
(2)Pseudogene because STOP-CODONS in J-REGION and defective RS
(3)STOP-CODON at position 1 (first 5' codon of germline J-REGION) may disappear during rearrangements
(4)Pseudogene because of frameshifts in J-REGION, defective RS and splice site
(5)noncanonical J-MOTIF
(6)Pseudogene because STOP-CODON in J-REGION


(B)


                                                                                        C-DOMAIN
                           A       AB      B           BC          C     CD      D          DE           E      EF   F          FG            G
                        (1-15)          (16-26)     (27-38)    (39-45)       (77-84)                 (85-96)     (97-104)  (105-117)    (118-128)
                    ——————————————>   ——————————>              ——————>       ———————>              ———————————>  ———————>  ——————————>  ——————————>
                    1        10  15   16     2326 27        38 3941 45       77 80 84              85  89    96  97   104 105       117 118121
            87654321|........|....|123|......|..| |..........| |.|...|1234567|..|...|12345677654321|...|......|12|......| |...........| |..|.....
   TRAC [EX1](X)IQDPDPAVYQLRDSK........SSDTTVCLYT DFGS.....QIN MTQSPAT.......DLKVFNSNSTVLDMG.AIGSKSNGVLSWSN........STSFGC QDAFSE...TFYA SE


                            CO                              TM            CY
                                                    |                   |
        [EX2] (E)FPCDPKLVEKSFET [EX3] (D)VSLNFQNLLVIVFRILLLKMVGFNLLMTLQLWSS
```
