# Supplementary material for: The Genomic Organisation of the TRA/TRD Locus Validates the Peculiar Characteristics of Dromedary δ-Chain Expression
Source: Genes (Basel). 2021 Apr 9;12(4):544. doi: 10.3390/genes12040544 (PMC8069558; doi:10.3390/genes12040544)
Supplement: Supplementary file 1 [file genes-12-00544-s001.zip › Supplementary files /Supplementary Figure S4.doc]

```
(A)

          5’D-NONAMER  12–spacer  5’D-HEPTAMER                 3’D-HEPTAMER      23-spacer        3’D-NONAMER
           GGTTTTTGT  ************  CACTGTG         TRDD         CACAGTG  ***********************  ACAAAAACC

  TRDD1    ggttattgt  actgctgtgttt  cactgtg    ACAGTATACGGAC     cacagtg  gggcaagtgtatgaagcctttgt  tcaaaaacc
                                               T  V  Y  G
                                                Q  Y  T  D
                                                 S  I  R

  TRDD2    ggtttttgt  aaaggtctgtag  cactgtg     GGTGGGATTTC      cacagtg  ctgcaaaacccacagagacctct  acagaaact
                                                G  G  I
                                                 V  G  F
                                                  W  D  F

  TRDD3    ggttattgt  actgctgtggtt  tactgtg      TCTCAGAAC       catagag  gttgacgtgcactaatcatttgt  tcaaaaacc
                                                 S  Q  N
                                                  L  R
                                                   S  E

  TRDD4    ggtttttgt  aaagctctgtag  cactgtg    ACTGGCTGGACTC     cacagtg  ctacaaaacccacagagacctct  acagaaact
                                               T  G  W  T
                                                L  A  G  L
                                                 W  L  D

  TRDD5    ggttattgt  actgctgtgttt  cactgtg    ACGATACGTGGAC     cacagtg  ggggaagtgtacgaagcctttgt  tcaaaaacc
                                               T  I  R  G
                                                R  Y  V  D
                                                 D  T  W

  TRDD6    ggtttttgt  aaagctctgtag  tactgtg     GCCAGGAGACT      cacagtg  atacaaaacccacagagacctgt  acaaaaact
                                                A  R  R
                                                 P  G  D
                                                  Q  E  T


 (B)

           J-NONAMER   12–spacer   J-HEPTAMER
           GGTTTTTGT  ************  CACTGTG                             TRDJ                            5'splice donor

  TRDJ1    ggtttttgg  aaagccctcaag  cactgtg  CTGTATCTACTAAAGGCAAGTTCGTCTTTGGAAATGGAATGCGTGTGATCGTGGAACCAA  gtaagt
                                               V  S  T  K  G  K  F  V  F  G  N  G  M  R  V  I  V  E  P

  TRDJ2    cttttttgt  aaggccatctca  agtggtg        ATTTGAAAGCACAGCTTATCTTTGGAAAGGGGACTCAACTCACCGTGGAACCAA  gtaagt
                                                     L  K  A  Q  L  I  F  G  K  G  T  Q  L  T  V  E  P

  TRDJ3    gttaccggt  aaggcagcacca  caatgtg   CTCCTGGGACACCCGACAGATGTTTTTTGGAGCTGGCACCAAACTCTTCGTGGAGCACC  gtgagt
                                               S  W  D  T  R  Q  M  F  F  G  A  G  T  K  L  F  V  E  H

  TRDJ4    agtttttag  actgggtttacc  agctgtg             CAGGATCCACTGATATTCGGCAAAGGAACCTATCTGAATGTTGAACCAC  gtaagt
                                                        Q  D  P  L  I  F  G  K  G  T  Y  L  N  V  E  P


(C)

                                                                                    C-DOMAIN
                           A       AB      B           BC        C     CD      D          DE           E      EF   F          FG            G
                        (1-15)          (16-26)     (27-38)  (39-45)       (77-84)                 (85-96)     (97-104)  (105-117)    (118-128)
                    ——————————————>   ——————————>            ——————>       ———————>              ———————————>  ———————>  ——————————>  ——————————>
                    1        10  15   16     2326 27      38 3941 45       77 80 84              85  89    96  97   104 105       117 118     128
            87654321|........|....|123|......|..| |........| |.|...|1234567|..|...|12345677654321|...|......|12|......| |...........| |.........|
TRDC   [EX1](X)KQSVATPSVFVMKNG...........TKVACLVK DFYP..KDIN INLQSAKKI.....KEYDPAIVVSPS.......GRYSAIKLGQYED......SDSVTC SVQHN...EQIFN STDLELKKTVS

                                   CO                                      TM          CY
                                                                  |                  |
       [EX2](V)TPKPKASENNNQTSKTCYEPQ          [EX3] (V)PAGKVNMMSLSVLGFRMLFAKTVAVNFLLTAKLFFF
```
