# Supplementary material for: The Genomic Organisation of the TRA/TRD Locus Validates the Peculiar Characteristics of Dromedary δ-Chain Expression
Source: Genes (Basel). 2021 Apr 9;12(4):544. doi: 10.3390/genes12040544 (PMC8069558; doi:10.3390/genes12040544)
Supplement: Supplementary file 1 [file genes-12-00544-s001.zip › Supplementary files /Supplementary Figure S2.doc]

```
                                 LEADER                     FR1-IMGT          CDR1-IMGT        FR2-IMGT      CDR2-IMGT                   FR3-IMGT                    CDR3-IMGT
                             		                                (1-26)            (27-38)         (39-55)        (56-65)                    (66-104)                    (105-117)
                                                        A             B           BC         C        C'        C'C"       C"         D           E          F          FG
                                                     (1-15)        (16-26)     (27-38)    (39-46)   (47-55)   (56-65)    (66-74)   (75-84)     (85-96)    (97-104)   (105-117)
                                                 ——————————————> ——————————>              ———————> ————————>            ————————> —————————> ———————————> ———————>
 Gene      Functionality                         1        10  15 16    23 26 27        38 3941  46 47     55 56      65 66     74 75   80 84 85  89    96 97   104 105
                                                 |........|....| |......|..| |..........| |.|....| |.......| |........| |.......| |....|...| |...|......| |......| |.....
TRDV1-1          F          MLLSSLLGVFLALIFSGSAV AQTVTQDQPAVSSHV WETVTLNCRYE TSYS.....TYY IFWYKQPP SGEMTFLIR QG.......S SSGNAKS.G RYSVNFRKAE KSLSLTISNLQL EDSAKYFC ALRE..[7.3.4]
TRDV1-2          F          MPLSSLLVVFLALIFSGSGV AQTVTQDQPAISSHV GETVTLNCRYE TSDS.....IYY IFWYKQPP SGEMTFLIH QG.......S SSGNANR.G RYSVNFRKAE KALSLTISNLLL KDSAKYFC ALRE..[7.3.4]
TRDV1-3          F          MPLSSLLGVFLALIFSGSGV AQTVTQDQPAISSHV GESATLNCRYE TSYS.....TYN IVWYKQPP SGEMTFLIH QG.......S SSGNAKS.G RYSINFRKAE KSLSLTISNLQL EDSAKYFC AFDG..[7.3.4]
TRDV1-5          F          MPLSSLLGVFLALVFSGSAV AQTVTQDQPALSSHL GESATLNCQYE TSYT.....EYY IFWYKQPP SGEMTFLIQ QY.......S SSGNAKS.G RYSVNFRKAE KSLSLTISNLQL EDSAKYFC ALEE..[7.3.4]
TRDV1-6          F          MPLSSLLGVFLALIFSGSAV AQTVTQDQPAVSSHV GQTATLNCQYE TSYS.....TYY IFWYKQPP SGEMTFLIR QE.......Y NRGNAKS.G RYSVNFRKAE KSLSLTISNLQL EDSAKYFC ALDE..[7.3.4]
TRDV1-7          F          MPLSSLLGVFLALIFSGSAV AQTVTQDQPAVSSHV GETATLNCRYE TSWS.....YYY IVWYKQPP SGEMTFLIH QD.......S SSGNAKS.G RYSINFRKAE KSLSLSISNLQL EDSAKYFC ALWE..[7.3.4]
TRDV2            F           MQRVCSLIHLTLFWAGIMS ADVLVPQDAAVTVSV GGSVTLRCSMK GGPI....SDYY NYWYRKIP GSTMTFVFR EG.......G TYGPGFQ.E NFRGEVDQVN NQVLLEILKASE GDKGSYYC AAT...[7.3.3]
TRDV3            F             MILLVGVSLLLACKGVL SAKVTQTSRDQTVAS GSEVTLPCTFE TEYS.....DPD LYWYRMRP GLSLEFVLY RDN....TES LNADFAQ.G RFSVQHNVAQ KTFHLVMSSVRA EDSATYYC VLEY..[7.6.4]
TRDV4            F           MLPTCPLFSLTIFCTGVSL GIILEPETKTLTVPL GGPATFRCNVT GGDL....GSYQ MSWYKKNE DQSLTSVYR QG.......N NATDGAR.S KFKGKVDALK SQYILDIQNATA EDVGTYYC GSDI..[7.3.4]
```
