# Supplementary material for: The Genomic Organisation of the TRA/TRD Locus Validates the Peculiar Characteristics of Dromedary δ-Chain Expression
Source: Genes (Basel). 2021 Apr 9;12(4):544. doi: 10.3390/genes12040544 (PMC8069558; doi:10.3390/genes12040544)
Supplement: Supplementary file 1 [file genes-12-00544-s001.zip › Supplementary files /Supplementary Table S1.doc]

**Supplementary Table S1.** Description of the related and not related TRA/TRD genes in the *Camelus dromedarius* chromosome 6 genome assembly (GeneBank ID: NC_044516.1). The position of all genes and their classification and functionality are reported.

| **Gene classification** | **Functionality** | **Position**^a^ |
| --- | --- | --- |
| OR10G3 | F | 31689543-31690499 |
| TRAV1 | F | 31696654-31697280 |
| OR4E2 | F | 31707436-31708362 |
| OR4E1 | F | 31717429-31718376 |
| TRAV2 | ORF^1^ | 31736701-31737199 |
| TRAV3 | F | 31742511-31742942 |
| TRAV4 | F | 31748120-31748874 |
| TRAV8-1 | ORF^2^ | 31754419-31754890 |
| TRAV9-1 | F | 31759532-31760017 |
| TRAV8-2 | P^3^ | 31764758-31765207 |
| TRAV43-1 | F | 31774473-31774989 |
| TRAV13-1 | P^4^ | 31781154-31781656 |
| TRAV14-1 | F | 31785864-31786384 |
| TRAV9-2 | F | 31792383-31792842 |
| TRAV13-2 | F | 31799671-31800178 |
| TRAV14-2 | P^5^ | 31804461-31804970 |
| TRAV9-3 | F | 31809647-31810106 |
| TRAV43-2 | P^6^ | 31818750-31819268 |
| TRAV13-3 | F | 31822066-31822556 |
| TRAV14-3 | P^4^ | 31828366-31827847 |
| TRAV14-4 | P^4^ | 31832156-31832809 |
| TRAV9-4 | F | 31836649-31837108 |
| TRAV12-1 | F | 31851091-31851621 |
| TRAV19-1 | P^5^ | 31858371-31857564 |
| TRAV45-1 | P^7^ | 31866227-31865756 |
| TRAV45-2 | P^7^ | 31871311-31871792 |
| TRAV16-1 | P^8^ | 31882954-31883430 |
| TRAV45-3 | P^9^ | 31891838-31892308 |
| TRAV45-4 | P^4^ | 31897992-31898431 |
| TRAV19-2 | P^4^ | 31899659-31900451 |
| TRAV22-1 | P^10^ | 31911608-31912112 |
| TRAV23-1 | P^9^ | 31915104-31915615 |
| TRAV24-1 | P^4^ | 31921561-31922066 |
| TRDV1-1 | F | 31929598-31930154 |
| TRAV23-2 | P^3^ | 31934245-31934754 |
| TRAV22-2 | F | 31938087-31938597 |
| TRAV19-3 | F | 31953976-31954762 |
| TRAV22-3 | F | 31966394-31966910 |
| TRAV23-3 | P^5^ | 31970628-31971133 |
| TRDV1-2 | F | 31975217-31975769 |
| TRAV24-2 | P^4^ | 31983315-31983820 |
| TRAV22-4 | P^5^ | 31993180-31993698 |
| TRAV45-5 | P^11^ | 32000820-32001299 |
| TRAV16-2 | P^5^ | 32004007-32004465 |
| TRAV12-2 | P^12^ | 32008697-32009209 |
| TRAV19-4 | P^8^ | 32016204-32016974 |
| TRAV45-6 | P^4^ | 32018212-32018660 |
| TRAV45-7 | P^4^ | 32024308-32024777 |
| TRAV45-8 | F | 32029812-32030284 |
| TRAV18 | F | 32035236-32035811 |
| TRAV17 | P^13^ | 32040842-32041356 |
| TRAV9-5 | P^1^ | 32073880-32074337 |
| TRAV44-1 | F | 32077906-32078677 |
| TRAV25-1 | P^4^ | 32083489-32084152 |
| TRAV24-3 | F | 32087191-32087697 |
| TRDV1-3 | F | 32094909-32095461 |
| TRAV23-4 | F | 32100999-32101501 |
| TRAV16-3 | P^14^ | 32103506-32103780 |
| TRAV22-5 | P^15^ | 31908813-31909268 |
| TRAV22-6 | P^4^ | 32113907-32114396 |
| TRAV23-5 | P^4^ | 32117653-32118159 |
| TRDV1-4 | P^8^ | 32122151-32122687 |
| TRAV24-4 | P^4^ | 32130017-32130505 |
| TRAV25-2 | F | 32134590-32135181 |
| TRAV8-3 | P^4^ | 32146238-32146688 |
| TRAV22-7 | F | 32147766-32148267 |
| TRDV1-5 | F | 32161493-32162041 |
| TRAV25-3 | F | 32169657-32170243 |
| TRAV44-2 | F | 32173786-32174554 |
| TRAV22-8 | F | 32179516-32180029 |
| TRDV1-6 | F | 32187929-32188479 |
| TRAV24-5 | P^6^ | 32194977-32195483 |
| TRAV25-4 | F | 32198570-32199157 |
| TRAV16-4 | P^14^ | 32203448-32203725 |
| TRAV23-4D | F | 32205727-32206229 |
| TRDV1-7 | F | 32211523-32212075 |
| TRAV24-6 | P^6^ | 32219303-32219811 |
| TRAV23-6 | P^4^ | 32233333-32233831 |
| TRDV1-8 | P^5^ | 32239060-32239610 |
| TRAV24-7 | F | 32246740-32247258 |
| TRAV27 | P^16^ | 32254225-32254772 |
| TRAV28 | F | 32258946-32259502 |
| TRAV29 | P^17^ | 32260280-32260814 |
| TRAV31 | P^14^ | 32264876-32265157 |
| TRAV33-1/DV6-1 | F | 32267623-32268187 |
| TRAV26 | P^12^ | 32276067-32276861 |
| TRAV33-2/DV6-2 | F | 32282208-32282772 |
| TRAV35 | P^4^ | 32285721-32286302 |
| TRAV36 | F | 32289994-32290536 |
| TRAV37 | P^10^ | 32310771-32311296 |
| TRAV38 | F | 32313390-32313974 |
| TRAV39 | F | 32327510-32328008 |
| TRAV40 | F | 32340338-32340766 |
| TRAV41 | F | 32344206-32344730 |
| TRDV5 | P^2^ | 32365033-32365552 |
| TRDV4 | F | 32404462-32404968 |
| TRDV2 | F | 32424880-32425405 |
| TRDD1 | F | 32440759-32440771 |
| TRDD2 | F | 32450204-32450214 |
| TRDD3 | ORF | 32455571-32455646 |
| TRDD4 | F | 32460329-32460408 |
| TRDD5 | F | 32467097-32467176 |
| TRDD6 | F | 32473147-32473224 |
| TRDJ1 | F | 32474479-32474538 |
| TRDJ4 | F | 32479441-32479489 |
| TRDJ2 | F | 32480882-32480935 |
| TRDJ3 | F | 32483208-32483266 |
| TRDC | F | 32487183-32489222 |
| TRDV3 | F | 32492070-32491450 |
| TRAJ61 | P | 32498839-32498888 |
| TRAJ60 | F | 32499855-32499911 |
| TRAJ59 | ORF | 32500108-32500173 |
| TRAJ58 | F | 32501263-32501325 |
| TRAJ57 | F | 32502460-32502522 |
| TRAJ56 | F | 32503095-32503155 |
| TRAJ55 | P | 32504638-32504700 |
| TRAJ54 | F | 32505858-32505915 |
| TRAJ53 | F | 32506526-32506591 |
| TRAJ52 | F | 32509757-32509825 |
| TRAJ50 | F | 32512027-32512082 |
| TRAJ49 | F | 32512941-32513000 |
| TRAJ48 | F | 32513756-32513816 |
| TRAJ47 | F | 32515714-32515770 |
| TRAJ46 | F | 32516248-32516310 |
| TRAJ45 | F | 32516746-32516811 |
| TRAJ44 | F | 32517599-32517661 |
| TRAJ43 | F | 32518706-32518762 |
| TRAJ42 | F | 32519525-32519590 |
| TRAJ41 | F | 32520022-32520083 |
| TRAJ40 | F | 32521726-32521782 |
| TRAJ39 | F | 32523930-32523992 |
| TRAJ38 | F | 32524573-32524634 |
| TRAJ37 | F | 32526104-32526165 |
| TRAJ36 | F | 32526813-32526873 |
| TRAJ35 | F | 32528364-32528426 |
| TRAJ34 | F | 32529410-32529467 |
| TRAJ33 | F | 32530095-32530151 |
| TRAJ32 | F | 32530944-32531009 |
| TRAJ31 | F | 32532520-32532576 |
| TRAJ30 | P | 32534334-32534391 |
| TRAJ29 | F | 32535429-32535488 |
| TRAJ28 | F | 32536451-32536516 |
| TRAJ27 | F | 32537108-32537166 |
| TRAJ26 | F | 32539295-32539354 |
| TRAJ25 | F | 32539592-32539651 |
| TRAJ24 | F | 32540288-32540350 |
| TRAJ23 | F | 32540727-32540789 |
| TRAJ22 | F | 32542324-32542386 |
| TRAJ21 | F | 32543858-32543912 |
| TRAJ20 | F | 32544614-32544670 |
| TRAJ19 | P | 32545592-32545647 |
| TRAJ18 | F | 32545977-32546042 |
| TRAJ17 | F | 32547103-32547165 |
| TRAJ16 | F | 32548523-32548583 |
| TRAJ15 | F | 32549511-32549570 |
| TRAJ14 | P | 32550209-32550260 |
| TRAJ13 | F | 32550941-32551001 |
| TRAJ12 | F | 32551991-32552050 |
| TRAJ11 | F | 32552564-32552623 |
| TRAJ10 | F | 32553720-32553783 |
| TRAJ9 | F | 32555685-32555745 |
| TRAJ8 | F | 32556305-32556362 |
| TRAJ7 | F | 32557824-32557879 |
| TRAJ6 | F | 32558457-32558518 |
| TRAJ5 | F | 32559666-32559725 |
| TRAJ4 | F | 32563342-32563402 |
| TRAJ3 | F | 32564305-32564366 |
| TRAJ2 | F | 32564853-32564918 |
| TRAJ1 | F | 32565838-32565899 |
| TRAC | F | 32569624-32573734 |

^a^ L-PART1/ V-exon for TRV genes

^1^ noncanonical DONOR-SPLICE

^2^ noncanonical RS

^3^ STOP-CODON in L-PART1

^4^ frameshift in V-REGION

^5^ frameshift in L-PART1, frameshift in V-REGION

^6^ STOP-CODON in V-REGION

^7^ noncanonical DONOR-SPLICE, frameshift in V-REGION

^8^ no INIT-CODON, frameshift in V-REGION

^9^ STOP-CODON in L-PART1, frameshift in V-REGION

^10^ noncanonical DONOR-SPLICE and ACCEPTOR-SPLICE, frameshift in V-REGION, noncanonical RS

^11^ frameshift in L-PART1, noncanonical ACCEPTOR-SPLICE

^12^ frameshift in L-PART1

^13^ frameshift in V-REGION, noncanonical RS

^14^ no L-PART1, frameshift in V-REGION

^15^ noncanonical ACCEPTOR-SPLICE, sequence gap in V-REGION

^16^ noncanonical ACCEPTOR-SPLICE

^17^ noncanonical ACCEPTOR-SPLICE, frameshift in V-REGION
