# Supplementary material for: The Genomic Organisation of the TRA/TRD Locus Validates the Peculiar Characteristics of Dromedary δ-Chain Expression
Source: Genes (Basel). 2021 Apr 9;12(4):544. doi: 10.3390/genes12040544 (PMC8069558; doi:10.3390/genes12040544)
Supplement: Supplementary file 1 [file genes-12-00544-s001.zip › Supplementary files /Supplementary FIgure S1 .doc]

```
                                   LEADER                  FR1-IMGT          CDR1-IMGT       FR2-IMGT       CDR2-IMGT                   FR3-IMGT                    CDR3-IMGT
                             		                    (1-26)            (27-38)         (39-55)        (56-65)                    (66-104)                    (105-117)
                                                        A             B           BC         C        C'        C'C"       C"         D           E          F          FG
                                                     (1-15)        (16-26)     (27-38)    (39-46)   (47-55)   (56-65)    (66-74)   (75-84)     (85-96)    (97-104)   (105-117)
                                                 ——————————————> ——————————>              ———————> ————————>            ————————> —————————> ———————————> ———————>
 Gene      Functionality                         1        10  15 16    23 26 27        38 3941  46 47     55 56      65 66     74 75   80 84 85  89    96 97   104 105
                                                 |........|....| |......|..| |..........| |.|....| |.......| |........| |.......| |....|...| |...|......| |......| |.....
TRAV1            F            MWGFFLLYVSLTVGGTV  GQGVQQ.PTDLMAME GEFVQVNCTYQ TSR......FDG LSWYQQHD GRAPVFLSY NVL....DGW ERR.....G RFSSFLSRSD AHSYLLLKELRT EDSASYLC AVI...[6.6.3]
TRAV2           ORF        MALRSTLEVVRLGLLLIAES  KDQVFQ.PSTVVSSE GAMAEIYCNHS ISN......AYN FFWYLHFP GCAPRLLIQ GS......KP SHQ.....G RYNMTYER.. LSSSLLIPQVRL ADAAVYYC GLA...[6.4.3]
TRAV3            F          MASACISTLAILFTLSGLR  AQSATQLKEPITGAE GEPLTVKCAYS LSG......TPY RFWYVQYP SQGLQFLLK YVAG..DNLV KGN.....Y GFEAEFNKSQ TSFHLKKASVQG SDSALYFC AVT...[6.8.3]
TRAV4            F            MRQMTRVTVLLILGTLS  LAKTIQ.PVFIDSYE GQEVNIPCNHT NIAT.....SEY IFWYRQFP NQGPQFVIQ GYK.....TN AAN.....E VASLFIPTDR KSSTLSLPWATL RDTAVYYC IVSD..[7.5.4]
TRAV8-1         ORF          MSLVPVLVLMTLFALRGA  QSVTQPDRHGITVPE GARLELRCNYS SSY......PPS LFWYVWYP NQGLQLLLK YTSG..NNLV SGI.....K GFVAEFRRNE TSFHLKKTSAHW SDSAEYFC AVV...[6.8.3]
TRAV8-2          P           MLLLLISVLGMTFTLNA*  AQTVTQPDDHVLISE KASLELRCNYS YGA......SVY LFWYVQYP CQGLQVLLK YFSG..DPLV QGI.....K SFESEFKRSE SSFNLRKSSVHW SDSAEYFC AVS...[6.8.3]
TRAV9-1          F          MNSSPGIVIVLFLMFGGSN  EDSVSQTEGQVTPSE GDSLIVNCSYE TTQ......YPA LFWYVQYP GEGPQLLLK ASRD...NEM ASN.....K GFKATYHRKP NSFHLEKASVRG ADSAVYYC ALG...[6.7.3]
TRAV9-2          F          MNSSPGLVTVVLLMLGRTH  GDSVMQTEGPVTLSE TAALTINCSYS TTT......YPT LFWYVQYS GEGPQLLLK ASRD...KEK VLK.....K GFEAMHHREQ TSFHLEKASVQG ADSAVYYC ALT...[6.7.3]
TRAV9-3          F          MNSSPGLVTVVLLMLGRTR  GDSVTQTEGPVTLPE SAALTMNCNYS AAG......YPT LFWYVQYP GEGPQLLLK ASRD...KEK VLK.....K GFEATYHREQ TSFHLEKASVRG ADSAVYYC ALG...[6.7.3]
TRAV9-4          F          MNSSPGLVTVVLLMLGRTR  GDSVTQTEGPVTLPE SAALTMNCNYS AAG......YPT LFWYVQYP GEGPQLLLK ASRD...KEK VLK.....K GFEATYHREQ TSFHLEKASVRG ADSAVYYC ALT...[6.7.3]
TRAV12-1         F          MLKSSTGFLVTLWLQLTWM  WSRQKGAAQSLNVPE GATASLTCSYS NSN......SQN FMWYRQYS GRGPEWLMT FYP....NND KQE.....G RYTAQVNKSS RYVSLHIRDSQP SDSAAYLC AES...[6.6.3]
TRAV13-2         F        MKTPIGALIMFLWLQLHCVSQ  GENVKQSPPSLSVQE GNSSVITCTYT DSN......SDF FPWYKQEP GKGLRFLIS IRSN...KVK EED.....Q GLTVLLNKTA KHLSLHITATQA GDTAVYFC AAS...[6.7.3]
TRAV13-3         F        MKTPIGALIMFLWLQLDCVSR  GENVEQSPPSLSVQE GNSSVITCTYT DSR......SEY FPWYKQEP GKGLRFLIS IRSN...KVK EED.....Q GLTVLLNKTA KHLSLHITATQA GDAAVYFC AAS...[6.7.3]
TRAV14-1         F         MLLSSLLKVAMASCWLGSSI  AQTVTQEQPPMLVQE KEAVTLDCTYD TSDP.....TYS LSWYKQPS SGAMTFLIR QDSY..NQQN ATE.....G HYSLNFQKAS KSIKLVISASQL EDSAVYFC ALSE..[7.8.4]
TRAV18           F          MLSASCSGLVILLILRGTS  GDTVTQTEDPVILPE GAFLTLNCTYE TSY......SAF LFWYVQYL NKEPELLLK SSE....NQK VVR.....G GFQATLVKSS RTFHLQKASVQT SDSAVYYC ALS...[6.6.3]
TRAV19-3         F         MRFPGLLRVVIASVCLGSVK  AQKVTQDQPAISRPE KEAATLDCVYE AKGY.....SYY LLWYKQAA SGEMLFLIH QESY..NERN TTE.....G RYFLNFQKSA SSISLTITALEL GDSAVYFC ALND..[7.8.4]
TRAV22-2         F         MKRLVGAVLGLLFAQICCVT  GEDVEQSPPVLSLQE GAASTLWCKFS SSV.......TS VHWYHQNP GGRLIHLFS VAS.....GT KHD.....G RFSATTVAKE RRSSLNISSSQT TDSVTYFC AV....[5.5.2]
TRAV22-3         F         MKRLVDAVLGLLFAQVCCVT  GEDVEQSPPVLSLQE GAASTLWCKFS SSV.......TS VHWYHQNP GGRLIHLFS VAS.....GT KHD.....G RFSATTVAKE RRSILNISSSQT TDSATYFC AVE...[5.5.3]
TRAV22-7         F         MKRLVGAVLGLLFAQVCCVT  GEDVEQSPPVLILQE GATSTLWCKFS SSV.......TS VHWYHQNP GGRLIHLFY VAS.....GT KHD.....G RFSATTVAKT ....VAASSSQT TDSATYFC AV....[5.5.2]
TRAV22-8         F         MKRLVGAVLGLLCAQVCCVT  GEDVEQSPPVLSLQE GATSTLWCKFS SSV.......TS LRWYRQNP GGRLIHLFY VAS.....GT KHD.....G RFSATTVIQE RRSSLNISSSQT TDSATYFC AV....[5.5.2]
TRAV23-2         P MDKLLGAVFLIPWLQVS*VSGQQNGKSE  QQQVKQSPQPLAVHE GAISILKCTYE NDA......FDY FPRYRQYP GQGPEFLIA KTSG...GNK KED.....G RFTVSSNKSA KHFSLHIKDSQP GDSATDFC AAS...[6.7.3]
TRAV23-4         F MDRILGASFLILWLQPGWVSGQQDGKSD  QQQVKQSPQPLEVHE GAISILNCTYE NSL......FNY FSWYRQYP GQGPEFLIG KSSG...GNK KED.....G RFTVSSNGNA KHFSLHIKDSQP GDAATYFC AAS...[6.7.3]
TRAV23-4D        F MDRILGASFLILWLQPGWVSGQQDGKSD  QQQVKQSPQPLEVHE GAISILNCTYE NSL......FNY FSWYRQYP GQGPEFLIG KSSG...GNK KED.....G.RFTVSSNGNA.KHFSLHIKDSQP GDAATYFC AAS...[6.7.3]
TRAV24-3         F        MERNPWPVSLLILWLHLGGSR  LLSMKQGPPRLHFQE GECTNATCSFP SSS......FYA FHWYRWEP ARSPKNLFV MSLN...GDE KEE.....G QVRVTLSTKD GHSSLHIKGPQP EDSAASLC AS....[6.7.2]
TRAV24-5         P        MERNPWPVSLLILWLHLRGSR  LLSTKQGPPRVHVQE GESTNATCSFP SSL......F*A FHCYRWEP ARSPKNLFV MSLN...GDE KEE.....G QVRVTLNTKD GHSSLHIKGPQP EDSATSLC AS....[5.7.2]
TRAV24-6         P        MERNPWPVSLLILWLHLGGGA AS*ARNRVLHGCTVQE GESINATCSFP SSS......FYA SHWYRWEP ARSPKNLFV MSLN...GDE KEE.....G QVRVTLSTKD SHSSLHIKGPQP EDSAASLC AS....[6.7.2]
TRAV24-7         F        MERNPWPVSCDLWLILVGGSR  LLSTKQGPPRVHVQE GESINATCSFP SSS......FYA SHWYRWEP ARSPKNLFV MSLN...GDE KEE.....G QVRVTLSTKD GHSSLHIKGPQP EDSAASLC AS....[6.7.2]
TRAV25-2         F           MFLITTILILWMQISQVN  GQQLKQIPEFVPLQE GENFTTYCNTS SPL.......DS LHWYKQQP GGRPVFLML LTKG...GDV KRP.....E RLTARFGETR KDRFLHIPAAQT ADVGNYFC AG....[5.7.2]
TRAV25-3         F           MFLITTILILWMQRPQVN  GQQLKQIPEFVPLQE GENFTTYCNTS SSL.......TS LQWYKQQP GGRPVFLMT LTKS...GDV TRQ.....G RLTARFGETR KDSFLHIPAAQT ADVGTYFC AG....[5.7.2]
TRAV25-4         F           MFLITTILILWMQISQVN  GQQLKQIPEFVPLQE GENFTTYCNTT SPL.......NS LQWYKQQP GGRPVFLMT LTKS...GDV TRQ.....G RLTARFGETR KDSFLHIPAAQT ADVGTYFC AG....[5.7.2]
TRAV27           P           MFLNFSVLVLWIQLAWES  TQQLEQSPRFLSVQE GENFTMYCNSS STF.......SS FHWYRQSP EEGPVLLMI LTKG...GEM KTQ.....K RLKARFGEAR KDSSLSSTAAQP GDAGVYLC AG....[5.7.2]
TRAV28           F        MKTQIGVLLGLLWIQIYWLRV  QMKVEQSPGVLTLQE GENSSLTCNFS VSL.......TS LQWFQQKP DGRLISLFY IAS.....GM.KQN.....G.RLTATVNIRE SYSHLHIRDSQP GDSATYFC AVE...[5.5.3]
TRAV33-1/DV6-1   F         MLLPGLLWAFVASFGFGSGM  AAKVTQNQTTETAQE GQAVTMHCTYE TTAQ.....RYT LYWYKQGP SGGMAFLIY QDDN...KAN AKR.....D RYSVNFQKAN QSISLTISSSQL ADSGKYFC ALWRY.[7.7.5]
TRAV33-2/DV6-2   F         MLLPGLLWAFVASFGFGSGM  AAKVTQTQTTETAQE GQAVTMHCTYE TSAL.....RYT LYWYKQGP SGGMAFLIY QDDN...KAN AKR.....D RYSVNFQKAK QSISLTISSSQL ADSGKYFC ALWDY.[7.7.5]
TRAV36           F        MEKWPQASLLIFWLQLSWVSS  DDKVIQSPPSLVVHE GTSATLNCSYK VTN......FQS LYWYKQEE KGL.AFLFV LVST...GIE KKS.....G RLRGTLDKKE LLRTLHITATEP EDSATYLC AVE...[6.7.3]
TRAV38           F         MTPVSLLWAALLSTCLGASV  AQMVTQPQPEVSVRE AQTATLDCTYN TVQS.....DYY LFWYKQPP SGETIFIIH QEAY..KQQN ATA.....N RYSVNFQKAA KSFSLRISDSQL EDAAMYFC AYTG..[7.8.4]
TRAV39           F           MKKLLAVILWLQLDWLNA  EVNVEQSPPSLSIQE GEDCTVYCNYS SGT......SDR LHWYRQDL GKSLEFLFL LTS....GAV KQE.....G QLTASLDTKA RRSTLHITASQP SFSATYFC ALD...[6.6.3]
TRAV40           F            MNSPLGSLILIFVFGGT  SSNSVQQTSQVTVSE GTSVTMNCTYT SAG......YPT LFWYVQYP NKALRFLQE ET......MK NSK.....N FGARNIKD.. KSSPITIYPAQI ADSAVYYC LLR...[6.4.3]
TRAV41           F        MGKPQQVLLIVLWLQLSWISG  ENEVEQSPPYLSAQE GDFVTVNCSYS VVM.......TT LQWLQQKP GGGIESLFI LSL.....EM KKE     G RVSATINRKE RHGSLHITAAQP RDSAVYIC AVE...[5.5.3]
TRAV43-1         F         MKRHWGSLLGLLWVQVCWVR  GMNVEQSPSALSIQE GTSSTLQCNFS NPV.......NY VQWFRQNP GGGLINLFY ITL.....GM KQN.....G RLNSTLNSKE QYSTLHITASQL EDSATYLC AAE...[5.5.3]
TRAV43-2         P         MKRHRGSLLGLLWLQISWVR  GVNVEQSPSALSLQE GTSSTLRCNFS AAV.......NG V*WFRQNP GGGLISLFY IAS.....GM KQN.....G RLNSTLNSKE RYSSLHITASQL EDSATYLC AAE...[5.5.3]
TRAV44-1         F            MRLVTGVAVLLTLGIVI  EAEIDQ.PKSKDFAE GEDVNLPCNHS TIGV.....SDY IHWYQQKP SQSPQYIIH GFR.....DT VTN.....N MASLTIATDR KSSTLVLPRVTL RDSAVYYC ALRE..[7.5.4]
TRAV44-2         F            MRLVTGVAVLLTWGTVI  EAAIEQ.PKSKDFTE GEDVTLHCNHS TIGL.....KDY IHWYWQNP NQSPQYIIH GLQ.....DT VTN.....N MASLTIATDR KSSSLVLPRVAL RDSAVYYC ALTE..[7.5.4]
TRAV45-8         F          MLSVSHLLLVIFLLYRGTS  GDSVTQTEVPVTLSE RELLMLNCTYQ TTDS.....DPY LFWYVQYV NKAPQLLLK GSAA...NPR PEH.....Q GFQATLVKSS RTFHLQKPSVQT SDSAVYYC ALR...[7.7.3]
```
